# Context-Dependent Associations of the *APOA1* rs670 Polymorphism with Apolipoprotein A1 and HDL Cholesterol: A Systematic Review and Meta-Analysis

**DOI:** 10.3390/ijms27177622

**Published:** 2026-08-25

**Authors:** Chien-Yi Chan, Yi-Chen Huang, Ming-Fen Lee, Chun-Yin Huang, Wen-Chieh Wu

**Affiliations:** 1 Department of Nutrition and Health Sciences, Chang Jung Christian University, Tainan 711301, Taiwan; wang731120@mail.cjcu.edu.tw; 2 Department of Nutrition, China Medical University, Taichung 406040, Taiwan; yichenhuang@mail.cmu.edu.tw (Y.-C.H.); leemf@mail.cmu.edu.tw (M.-F.L.)

**Keywords:** *APOA1* rs670, meta-analysis, HDL cholesterol, apolipoprotein A1, metabolic syndrome, polymorphism

## Abstract

The *APOA1* rs670 (−75 G>A) polymorphism has been implicated in lipid metabolism and susceptibility to metabolic syndrome (MetS). However, individual studies have reported inconsistent associations with circulating apolipoprotein A1 (Apo A1) and lipid parameters, and no meta-analysis has comprehensively evaluated these associations stratified by metabolic disorder status. We therefore quantified the associations between *APOA1* rs670 and serum Apo A1 and lipid parameters, and determined whether these associations are modified by metabolic disorder status. PubMed, Embase, Web of Science, Cochrane Library, and ClinicalTrials.gov were searched from inception to March 2026. Eligible studies reported *APOA1* rs670 genotype data with quantitative lipid measurements. Forty studies involving 22,175 participants met the inclusion criteria. Pooled effect sizes were estimated as standardized mean differences (SMDs) with 95% confidence intervals (CIs) using random-effects models. This review was registered in PROSPERO (CRD420261333145). A allele carriers had significantly higher serum Apo A1 (SMD, 0.20; *p* < 0.001) and HDL-C levels (SMD, 0.14; *p* < 0.001) than GG homozygotes. Subgroup analyses stratified by metabolic disorder status revealed that the associations with Apo A1 (SMD, 0.53 vs. 0.11) and HDL-C (SMD, 0.23 vs. 0.08) were substantially stronger among individuals with metabolic disorders than among metabolically healthy individuals. No significant associations were observed for LDL-C or triglyceride levels. The *APOA1* rs670 polymorphism is associated with higher circulating Apo A1 and HDL-C levels, with stronger associations in individuals with metabolic disorders. These findings suggest that metabolic status modifies the phenotypic effects of rs670 on the Apo A1–HDL pathway.

## 1. Introduction

The global burden of metabolic syndrome (MetS) has increased markedly over recent decades, largely driven by environmental and demographic transitions [1,2]. MetS is characterized by a cluster of metabolically interrelated risk factors, including abdominal adiposity, hypertension, hyperglycemia, hypertriglyceridemia, and reduced high-density lipoprotein cholesterol (HDL-C) levels [3]. Collectively, these abnormalities substantially elevate the risk of cardiovascular disease, type 2 diabetes mellitus, and premature mortality [4]. Although lifestyle factors play a critical role in the development of MetS, accumulating evidence suggests that genetic variation also contributes significantly to interindividual susceptibility to metabolic dysfunction.

Apolipoprotein A1 (Apo A1), encoded by *APOA1* gene, is the principal protein component of HDL particles and plays a crucial role in reverse cholesterol transport by facilitating the efflux of cholesterol from peripheral tissues to the liver for metabolism and excretion [5,6,7]. Variants within the *APOA1* locus have therefore attracted considerable interest as potential determinants of lipid metabolism and cardiometabolic risk. The *APOA1* rs670 (−75 G>A) single-nucleotide polymorphism, located in the promoter region of the gene on chromosome 11q23, has been proposed to influence transcriptional activity and circulating lipid levels [8,9]. The A allele frequency of rs670 varies across ethnic populations. According to the 1000 Genomes Project (*n* = 5008), the global A allele frequency is approximately 18.9%, ranging from 15.4% in European populations to 24.3% in East Asian populations, with intermediate frequencies observed in South Asian (18.9%), American (21.2%), and African (16.0%) populations [10]. This variation in allele frequency may partly contribute to the heterogeneity observed across association studies. Functional evidence suggests that the A allele may enhance *APOA1* gene expression, potentially resulting in higher Apo A1 and HDL-C concentrations than the G allele [11,12,13].

Several epidemiological studies have explored the association between the rs670 polymorphism and lipid traits or metabolic outcomes. Some investigations have reported that the A allele is associated with favorable metabolic profiles, including elevated HDL-C levels and improved insulin sensitivity, particularly among healthy individuals or those on energy-restricted diets [14,15]. However, findings remain inconsistent across populations. While certain studies suggest a protective role of rs670 against MetS and dyslipidemia, others have reported null or even opposing associations in individuals with established metabolic diseases such as coronary artery disease or diabetes [16,17,18]. These discrepancies may reflect differences in population genetics, environmental exposures, dietary patterns, sex-specific effects, and underlying metabolic status.

Importantly, most existing studies have examined rs670 associations within relatively homogeneous populations without explicitly considering metabolic context as a potential effect modifier. Consequently, it remains unclear whether the influence of rs670 on lipid metabolism differs between metabolically healthy individuals and those with metabolic disorders. Addressing this gap is critical for understanding the context-dependent role of *APOA1* genetic variation and its potential relevance to precision medicine approaches targeting metabolic diseases. Therefore, we conducted a systematic review and meta-analysis to assess the pooled associations of *APOA1* rs670 with circulating Apo A1 and lipid parameters, and to determine whether metabolic disorder status modifies these associations.

## 2. Results

### 2.1. Study Characteristics

**Figure 1:**
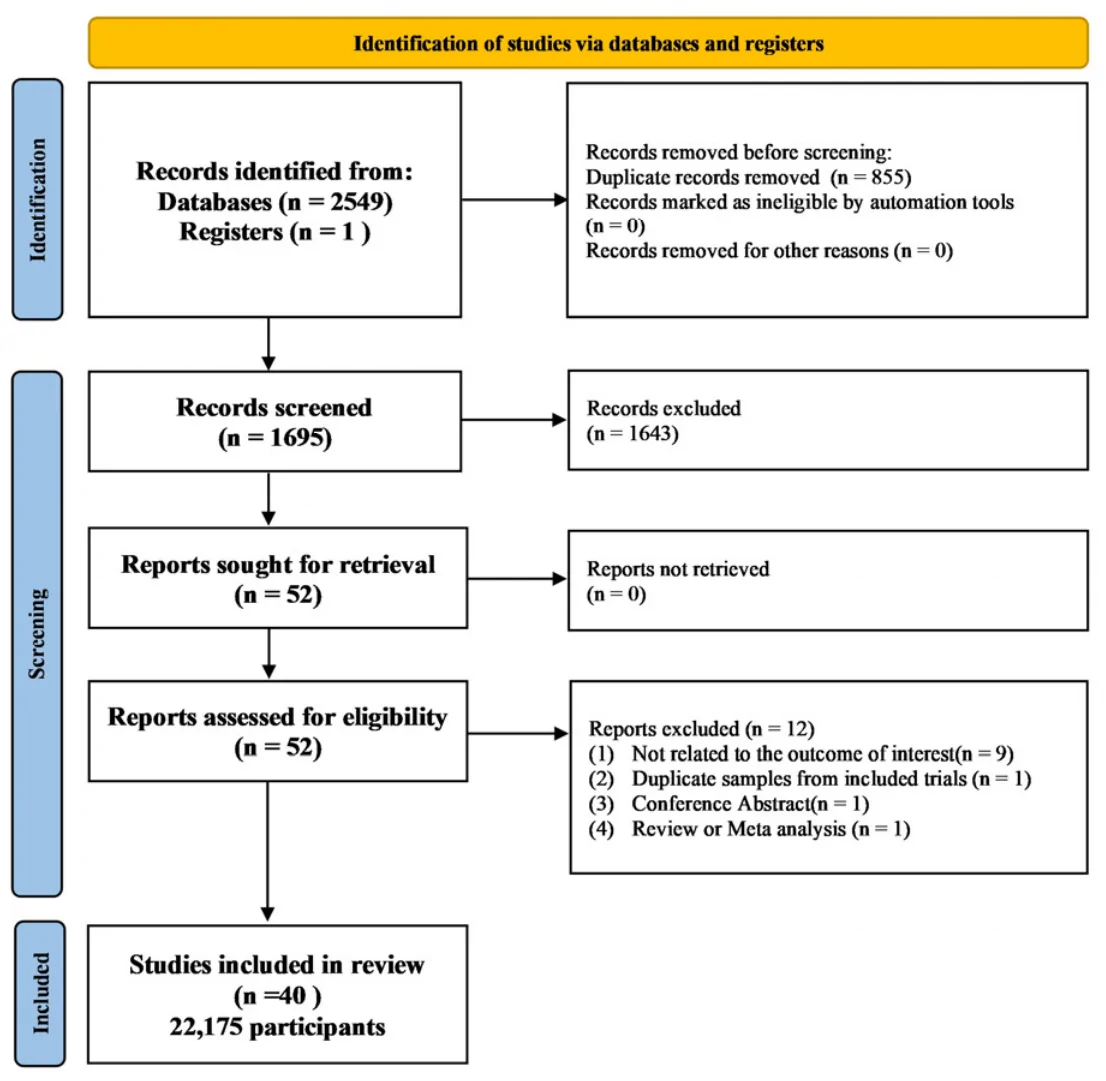
PRISMA flow diagram of the study selection process for the systematic review and meta-analysis.

During the initial database search, 2550 records were identified from PubMed, Embase, Web of Science, the Cochrane Library, and ClinicalTrials.gov. After removal of 855 duplicate records, 1695 studies remained for title and abstract screening; 1643 were excluded for not meeting the inclusion criteria. The full texts of 52 studies were assessed, of which 12 were excluded due to irrelevant outcomes or duplicate samples. Ultimately, 40 studies were included (Figure 1).

Detailed characteristics are summarized in Supplementary Table S3. The 40 publications (1990–2026) comprised cross-sectional, case–control, cohort, and randomized clinical trial designs, with 22,175 participants from Asia [19,20,21,22,23,24,25,26,27,28,29,30,31,32,33,34,35], the Middle East [36,37,38], Europe [11,12,13,16,39,40,41,42,43,44,45,46], South America [47,48,49], the USA [15,50,51], and Australia [52,53].

### 2.2. Association Between the APOA1 rs670 Polymorphism and Serum Apo A1 Levels

**Figure 2:**
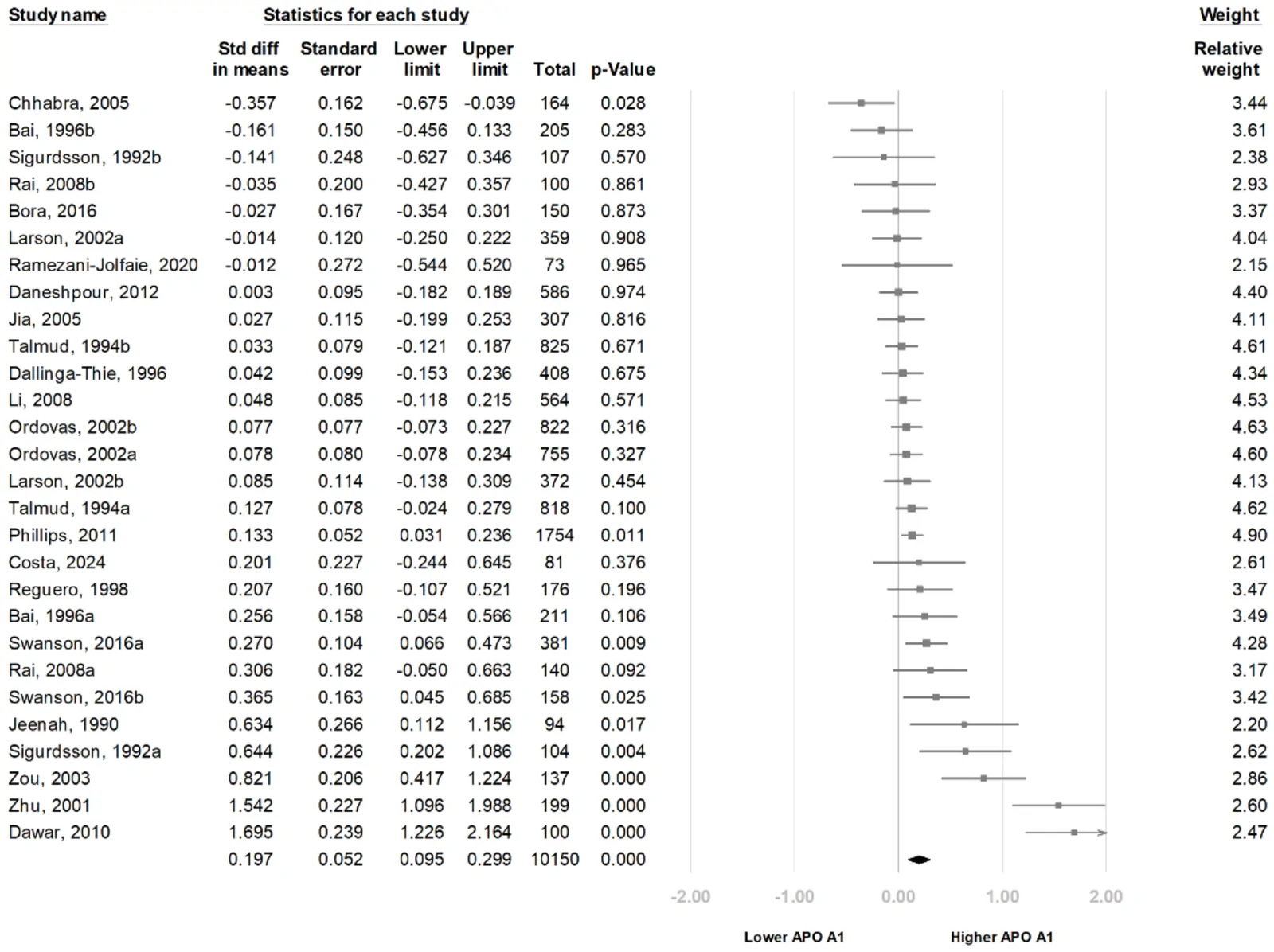
Forest plot showing the standardized mean differences (SMDs) in serum Apo A1 levels between *APOA1* rs670 A allele carriers (GA/AA genotypes) and GG homozygotes. Each study is represented by its effect size with 95% confidence intervals (CIs), and the size of the squares reflects the relative weight of each study in the meta-analysis [11,12,13,15,16,19,21,22,26,27,29,32,33,34,36,38,39,40,49,50,51].

Pooled analysis of the included studies showed that carriers of the *APOA1* rs670 A allele (GA or AA genotypes) had significantly higher serum Apo A1 levels than individuals carrying the GG genotype (Figure 2; SMD, 0.197; 95% CI, 0.095–0.299; *p* < 0.001; I^2^ = 79.4%).

**Figure 3:**
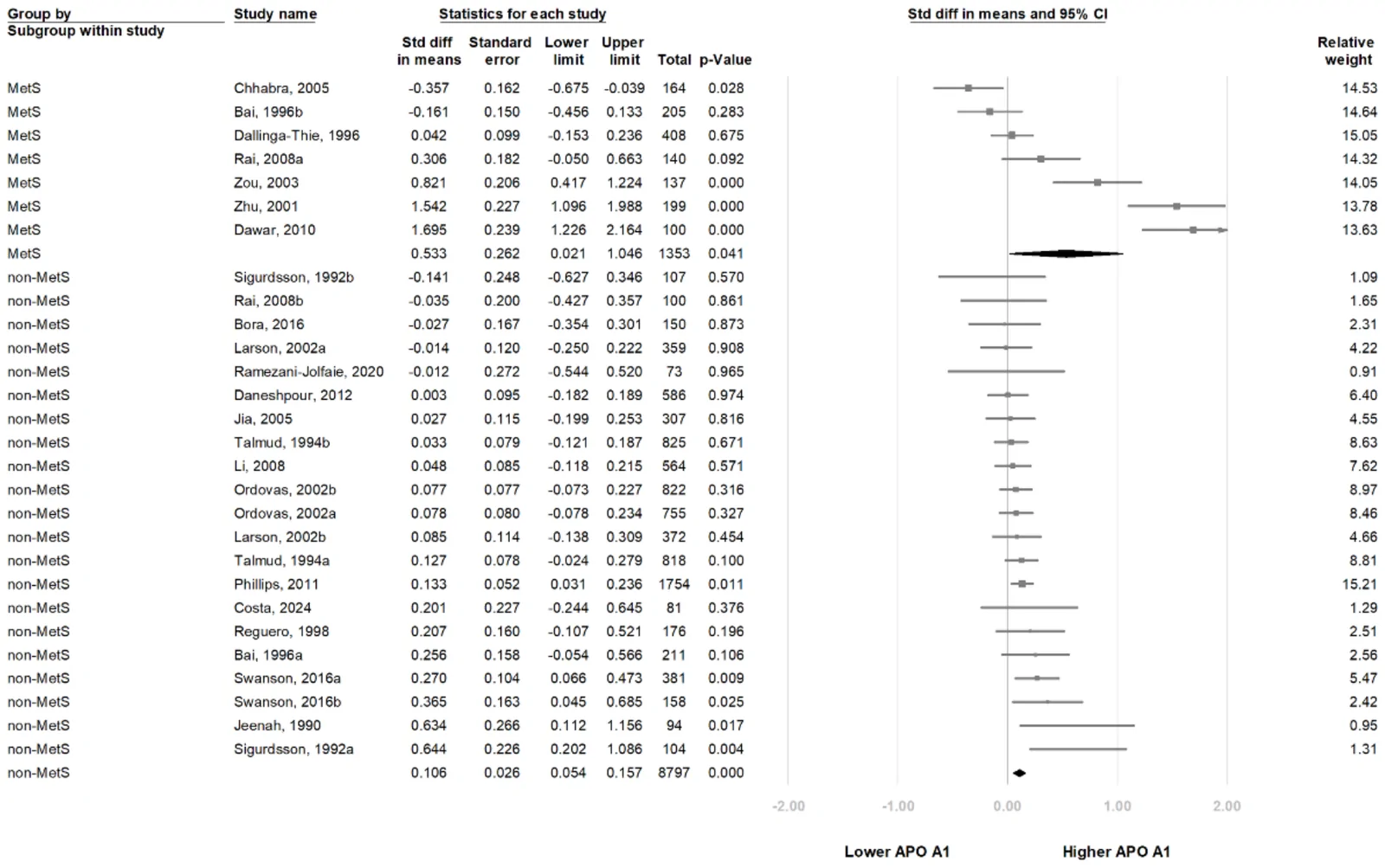
Forest plot of standardized mean differences (SMDs) in serum Apo A1 levels between *APOA1* rs670 A allele carriers (GA/AA genotypes) and GG homozygotes, stratified by metabolic status (MetS vs. non-MetS). Each study is presented with its effect size and corresponding 95% confidence interval (CI), and the size of each square reflects the relative weight of the study in the meta-analysis [11,12,13,15,16,19,21,22,26,27,29,32,33,34,36,38,39,40,49,50,51].

To explore whether metabolic status influenced this association, subgroup analyses were conducted stratifying participants into MetS and non-MetS groups (Figure 3). The results demonstrated that A allele carriers had significantly higher Apo A1 levels in both individuals with metabolic disorders (SMD, 0.533; 95% CI, 0.021–1.046; *p* = 0.041; I^2^ = 94.1%) and those without metabolic disorders (SMD, 0.106; 95% CI, 0.054–0.157; *p* < 0.001; I^2^ = 13.1%). Notably, the magnitude of the association was substantially greater in individuals with metabolic disorders, with the effect size for Apo A1 elevation being approximately fivefold larger than that observed in metabolically healthy individuals.

Sensitivity analysis using the leave-one-out method confirmed the robustness of the overall association between the rs670 A allele and elevated serum Apo A1 levels (Supplementary Figure S1a).

### 2.3. Association Between the APOA1 rs670 Polymorphism and Metabolic Syndrome-Related Lipid Parameters

**Figure 4:**
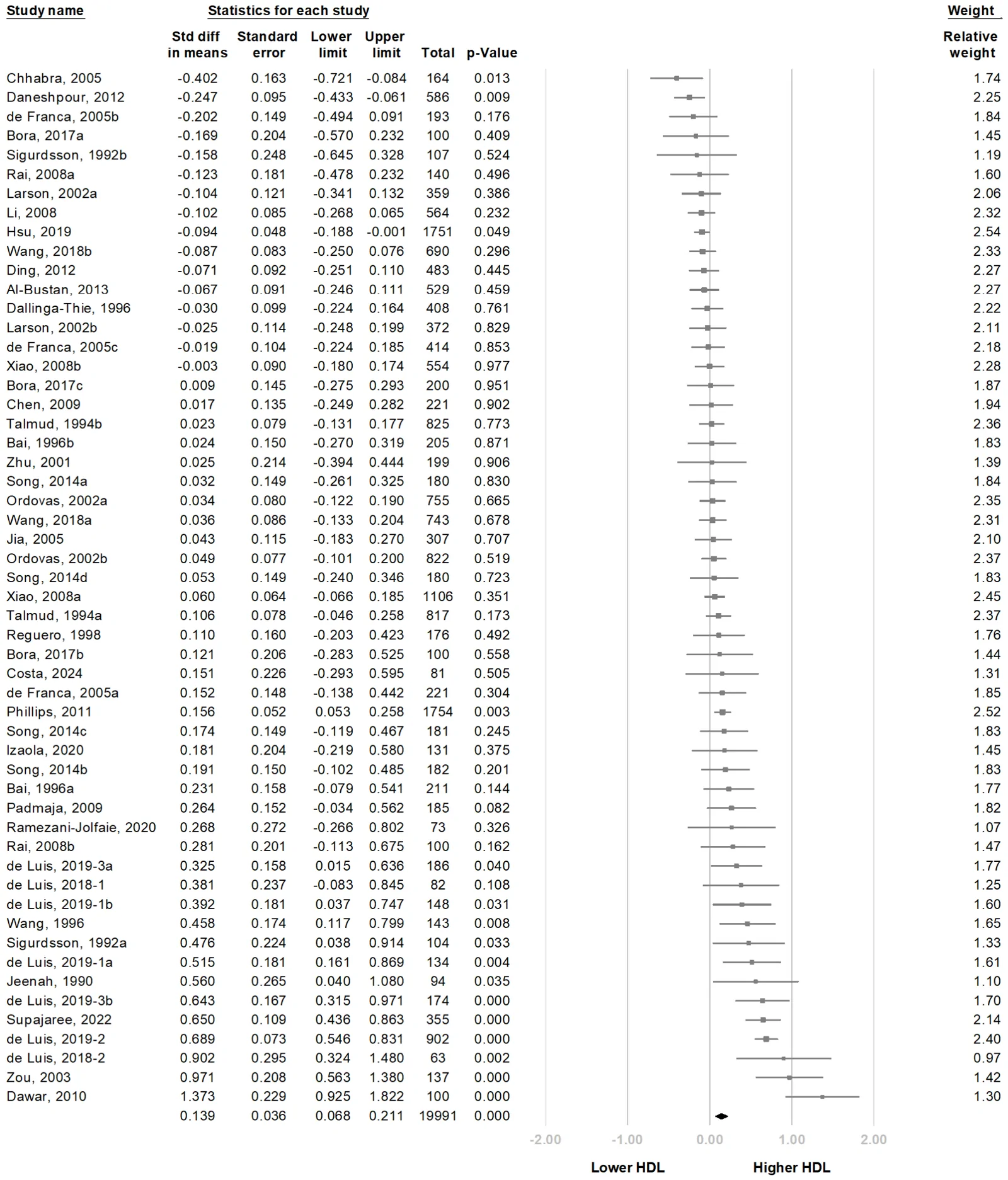
Forest plot of standardized mean differences (SMDs) in serum HDL-C levels between *APOA1* rs670 A allele carriers (GA/AA genotypes) and GG homozygotes. Each study is presented with its effect size and corresponding 95% confidence interval (CI), and the size of the squares reflects the relative weight of each study in the meta-analysis [11,12,13,15,16,19,20,21,22,23,24,25,26,27,28,29,30,31,32,33,36,37,38,39,40,41,42,43,44,45,46,47,48,49,50,52,53].

Overall pooled analysis showed that individuals carrying the *APOA1* rs670 GA or AA genotype had significantly higher HDL-C levels than those with the GG genotype (Figure 4; SMD, 0.139; 95% CI, 0.068–0.211; *p* < 0.001; I^2^ = 79.7%).

No statistically significant association was observed between the rs670 GA or AA genotype and LDL-C levels (Supplementary Figure S2a; SMD, 0.069; 95% CI, −0.003–0.141; *p* = 0.059; I^2^ = 76.0%). A modest but statistically significant positive association was identified for total cholesterol (TC) (Supplementary Figure S2b; SMD, 0.082; 95% CI, 0.013–0.150; *p* = 0.019; I^2^ = 66.8%), whereas no significant association was detected for triglyceride (TG) levels (Supplementary Figure S2c; SMD, −0.035; 95% CI, −0.109–0.039; *p* = 0.352; I^2^ = 78.4%).

**Figure 5:**
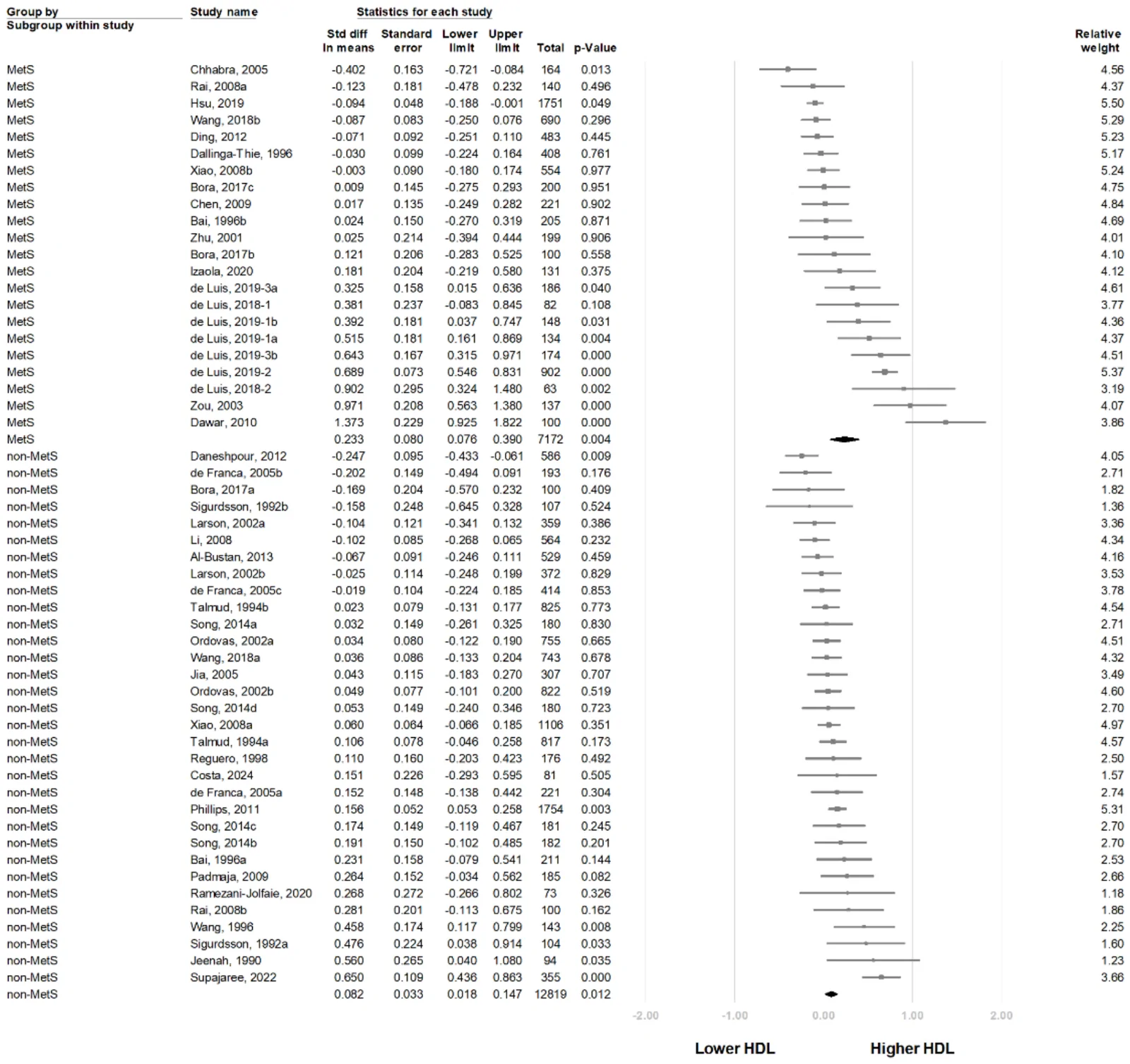
Forest plot of standardized mean differences (SMDs) in serum HDL-C levels between *APOA1* rs670 A allele carriers (GA/AA genotypes) and GG homozygotes, stratified by metabolic status (MetS vs. non-MetS). Each study is presented with its effect size and corresponding 95% confidence interval (CI), and the size of each square reflects the relative weight of the study in the meta-analysis [11,12,13,15,16,19,20,21,22,23,24,25,26,27,28,29,30,31,32,33,36,37,38,39,40,41,42,43,44,45,46,47,48,49,50,52,53].

Subgroup analyses stratified by metabolic status revealed that the rs670 GA or AA genotype was consistently associated with higher HDL-C levels in both individuals with metabolic disorders (SMD, 0.233; 95% CI, 0.076–0.390; *p* = 0.004; I^2^ = 88.5%) and those without metabolic disorders (SMD, 0.082; 95% CI, 0.018–0.147; *p* = 0.012; I^2^ = 59.4%) (Figure 5). The effect size was notably larger in the metabolic disorders subgroup.

No significant associations were observed between the rs670 GA or AA genotype and LDL-C levels in either the metabolic disorders subgroup (SMD, 0.075; 95% CI, −0.070–0.219; *p* = 0.310; I^2^ = 83.8%) or the non-metabolic disorders subgroup (SMD, 0.073; 95% CI, −0.001–0.146; *p* = 0.053; I^2^ = 63.0%) (Supplementary Figure S3a).

Similarly, TC levels were not significantly associated with the rs670 GA or AA genotype in individuals with metabolic disorders (SMD, 0.082; 95% CI, −0.074–0.237; *p* = 0.304; I^2^ = 84.7%), although a significant association was observed in metabolically healthy individuals (SMD, 0.085; 95% CI, 0.038–0.131; *p* < 0.001; I^2^ = 0%) (Supplementary Figure S3b). No significant associations were detected for TG levels in either subgroup (Supplementary Figure S3c).

Sensitivity analyses confirmed the stability of the observed association between the *APOA1* rs670 GA or AA genotype and HDL-C levels (Supplementary Figure S1b). The null associations for LDL-C (Supplementary Figure S1c) and TG levels (Supplementary Figure S1d) remained unchanged, while the modest positive association with TC was also stable (Supplementary Figure S1e).

### 2.4. Publication Bias

Potential publication bias was evaluated through visual inspection of funnel plots (Supplementary Figure S4a–e). Mild asymmetry was observed in the funnel plots for serum Apo A1 and HDL-C levels, suggesting a slight underrepresentation of smaller studies reporting null or negative findings.

This observation was supported by significant Begg’s rank correlation test results for these outcomes. In contrast, funnel plots for LDL-C, TC, and TG levels appeared largely symmetrical, with no statistical evidence of publication bias detected.

## 3. Discussion

### 3.1. Principal Findings and Effect Sizes

The present meta-analysis demonstrates that the *APOA1* rs670 polymorphism is significantly associated with serum Apo A1 and HDL-C levels, with the effect being particularly pronounced in individuals with metabolic disorders.

Carriers of the A allele (GA or AA genotypes) exhibited significantly higher serum Apo A1 and HDL-C levels than individuals carrying the GG genotype. Notably, the SMD for Apo A1 was approximately fivefold greater in participants with metabolic disorders than in those without (0.533 vs. 0.106), while the effect on HDL-C was nearly threefold larger (0.233 vs. 0.082). In contrast, no significant associations were observed for LDL-C, TC, or TGs, regardless of metabolic status. These findings suggest that rs670 is primarily associated with HDL-related metabolic pathways and may confer a protective effect against cardiometabolic risk, particularly under conditions of metabolic dysregulation.

Although the observed effect sizes for Apo A1 (SMD, 0.197) and HDL-C (SMD, 0.139) may appear modest, they are consistent with the polygenic architecture of lipid traits. Large-scale genome-wide association studies (GWASs), including those conducted by the Global Lipids Genetics Consortium (GLGC), have demonstrated that common lipid-associated variants typically exert small individual effects, generally ranging from 0.05 to 0.15 standard deviations [54,55]. Within this context, the magnitude of the rs670 effect is biologically plausible and clinically meaningful for a common genetic variant influencing lipid metabolism. Importantly, our subgroup analyses indicate that average GWAS-derived estimates may underestimate genetic effects in populations experiencing metabolic stress.

### 3.2. Mechanistic Basis and Metabolic Context

From a mechanistic perspective, the rs670 (−75 G>A) polymorphism is located within the promoter region of the *APOA1* gene, a critical regulatory site controlling transcriptional activity [8,9]. The present findings support the hypothesis that the A allele may be associated with enhanced *APOA1* transcription, resulting in increased circulating Apo A1 protein levels [11,12,13]. As Apo A1 is the principal structural and functional component of HDL particles and a key mediator of reverse cholesterol transport, elevated Apo A1 availability provides a plausible biological explanation for the higher HDL-C concentrations observed among A allele carriers [5,6,7].

The specificity of the rs670 effect to the Apo A1-HDL axis further supports its biological plausibility. A allele carriers consistently demonstrated higher serum Apo A1 and HDL-C levels, whereas associations with LDL-C and TGs were absent, and the modest association with TC likely reflects the contribution of HDL-C to total cholesterol levels. This pattern suggests that rs670 does not broadly influence lipid metabolism but instead selectively modulates HDL particle formation and cholesterol efflux pathways. Such specificity is consistent with previous genetic studies indicating that most lipid-associated loci influence distinct lipid fractions rather than all lipid parameters simultaneously [56].

A key contribution of the present study is the finding that the association between the *APOA1* rs670 polymorphism and lipid traits appears to be modified by metabolic status. The substantially larger effect sizes observed in individuals with metabolic disorders suggest the presence of gene–environment or gene–disease interactions, whereby genetic regulation of lipid metabolism may become more influential under conditions of insulin resistance, obesity, chronic inflammation, or oxidative stress. In these settings, alterations in transcriptional regulation may enhance the functional impact of promoter variants such as rs670, providing a plausible biological explanation for the amplified associations observed in metabolically compromised populations [49]. Such context-dependent effects may not be readily detected in population-wide genome-wide association studies (GWASs), which estimate average genetic effects across metabolically heterogeneous populations [55]. Nevertheless, this interpretation should be viewed with caution. The MetS subgroup exhibited substantial between-study heterogeneity (I^2^ = 94.1%) and included a relatively limited number of studies. Moreover, because this meta-analysis was based on subgroup comparisons rather than a formal test of interaction, the apparent effect modification by metabolic status should be considered hypothesis-generating and requires confirmation in larger, well-characterized cohorts before definitive conclusions can be drawn. The amplified associations observed in individuals with MetS may partly reflect quantile-dependent expressivity, whereby the phenotypic effects of the rs670 A allele appear more pronounced under conditions of metabolic dysregulation; individual participant data analyses would be needed to formally evaluate this possibility.

Beyond metabolic status, the phenotypic expression of rs670 may also be influenced by lifestyle factors. For example, smoking has been reported to attenuate or abolish the beneficial effect of the A allele on HDL-C levels [12,57]. Conversely, higher intake of polyunsaturated fatty acids (PUFAs) has been associated with enhanced HDL-C responses among A allele carriers, particularly under dietary interventions such as Mediterranean or low-fat diets [15,44,45]. These gene–environment interactions suggest that rs670 may function as a metabolic modifier, with its cardioprotective potential being most evident under favorable lifestyle conditions. These findings raise the possibility that rs670 A allele carriers may respond differently to lifestyle or pharmacological interventions, including dietary fat modification, structured physical activity, or statin therapy. Prospective genotype-stratified studies are needed to evaluate these possibilities.

Experimental studies further support the notion that promoter variants may exhibit context-dependent regulatory effects, as transcription factor binding and chromatin accessibility can vary according to cellular metabolic states [58,59]. Metabolic dysregulation can alter key lipid-regulating pathways, including liver X receptor (LXR) and peroxisome proliferator-activated receptor (PPAR) signaling [60]. Under such conditions, the rs670 A allele may exert stronger effects on *APOA1* transcription, providing a plausible mechanistic explanation for the amplified associations observed among individuals with metabolic disorders.

### 3.3. Study Limitations and Future Directions

Although the rs670 A allele is consistently associated with higher HDL-C levels in the present analysis, the clinical implications of these findings warrant careful consideration. Large-scale Mendelian randomization studies have failed to demonstrate a causal relationship between HDL-C and atherosclerotic cardiovascular disease, suggesting that HDL-C may serve as a marker rather than a mediator of cardiovascular risk [61]. Whether the rs670-associated elevation in Apo A1 and HDL-C translates into reduced atherosclerosis burden or CVD events remains to be established in prospective genotype-stratified studies.

The mild asymmetry observed in funnel plots for Apo A1 and HDL-C suggests the possibility of publication bias, potentially reflecting underrepresentation of smaller studies reporting null effects. However, funnel plot asymmetry may also arise from true heterogeneity across studies. In the present analysis, smaller studies were more likely to involve populations with greater metabolic dysregulation, where the genetic effect of rs670 appeared substantially amplified. Such context-dependent variation may contribute to the observed distribution of effect sizes without necessarily indicating selective reporting. Importantly, sensitivity analyses confirmed that the primary findings remained stable, supporting the robustness of the results.

Several limitations should be considered when interpreting the present findings. First, most included studies were conducted in Asian and European populations, potentially limiting the generalizability of the results to other ethnic groups with different allele frequencies, genetic backgrounds, and environmental exposures. Second, important environmental factors—including dietary patterns, smoking status, and physical activity—that may modify the effects of the *APOA1* rs670 polymorphism could not be systematically evaluated because these variables were inconsistently reported across the included studies. Third, this meta-analysis was based on aggregate study-level data, precluding direct assessment of individual-level gene–environment and gene–metabolic interactions. Fourth, substantial between-study heterogeneity was observed for several outcomes, particularly among participants with metabolic disorders, suggesting that unmeasured differences in study populations or methodologies may have influenced the pooled estimates. Fifth, the examination of atherogenic lipid ratios, including total cholesterol:HDL-C and LDL-C:HDL-C, was not feasible in the present analysis owing to insufficient availability of these data across the included studies. Future meta-analyses with access to more complete lipid profiles may address this limitation. Finally, the conversion of medians and interquartile ranges to estimated means and standard deviations in a subset of studies may have introduced minor imprecision into the calculated effect sizes.

Despite these limitations, the consistent positive associations observed for serum Apo A1 and HDL-C levels across diverse populations support a role for the *APOA1* rs670 A allele in modulating HDL-related lipid metabolism. Notably, the stronger associations observed among individuals with metabolic disorders suggest that the phenotypic effects of rs670 may depend on metabolic context. However, these subgroup findings should be considered exploratory, given the substantial heterogeneity and limited number of studies in the MetS subgroup. Whether *APOA1* rs670 genotyping provides clinically meaningful information beyond conventional lipid profiling and established cardiometabolic risk factors remains uncertain and should be evaluated in large, prospective studies with standardized metabolic phenotyping and comprehensive assessment of environmental exposures.

## 4. Materials and Methods

### 4.1. Study Registration

This meta-analysis was registered in the PROSPERO database (registration no. CRD420261333145) and conducted in accordance with the Preferred Reporting Items for Systematic Reviews and Meta-Analyses (PRISMA) 2020 guidelines (Supplementary Table S1) [62].

### 4.2. Search Strategy

A comprehensive literature search was performed in PubMed (NCBI), Embase (OVID), Web of Science (Clarivate Analytics), the Cochrane Library, and ClinicalTrials.gov to identify studies investigating the association between the *APOA1* rs670 polymorphism, serum Apo A1 levels, and lipid components related to metabolic syndrome. The search covered all records from database inception to March 2026. The search strategy combined Medical Subject Headings (MeSH) and free-text terms related to three domains: (1) Apolipoprotein A1 (APOA1, “apolipoprotein A-I”), (2) genetic variation (polymorphism, SNP, rs670, “−75 G>A”), and (3) metabolic syndrome or lipid traits (hypertriglyceridemia, low HDL-C, central obesity, hyperglycemia, hypertension). The detailed search strategy is provided in Supplementary Table S2. Additionally, reference lists of eligible articles, relevant review papers, and previous meta-analyses were manually screened to identify further potentially eligible studies. The study selection process is illustrated in Figure 1. Two reviewers (W-C W and C-Y C) independently screened titles and abstracts for eligibility.

### 4.3. Selection Criteria

Studies were considered eligible if they met the following criteria: (1) included human participants of any age or sex; (2) used cross-sectional, case–control, cohort, or randomized controlled trial designs; (3) reported *APOA1* rs670 genotype data (GG, GA, or AA) together with quantitative measurements of at least one lipid parameter, including serum Apo A1 concentration, HDL-C, LDL-C, total cholesterol (TC), or triglycerides (TGs); and (4) were full-text articles published in peer-reviewed journals, without language restrictions. Non-English articles were translated using Google Translate (https://translate.google.com, accessed March 2026) before data extraction.

Studies were excluded if they were conference abstracts, editorials, commentaries, letters, review articles, or study protocols, or if they lacked sufficient genotype information or quantitative lipid data required for meta-analysis.

### 4.4. Data Extraction

Data extraction was independently performed by two reviewers using a standardized data extraction form adapted from the Cochrane Collaboration guidelines. Discrepancies were resolved through discussion or consultation with a third reviewer. Extracted variables included the first author’s name, publication year, country or region, study design, sample size, participant characteristics, and *APOA1* rs670 genotype distribution (GG, GA, AA), as well as quantitative measures of serum Apo A1 concentration, HDL-C, LDL-C, TC, and TGs. When studies reported data from multiple independent subgroups (e.g., stratified by metabolic status, sex, or clinical characteristics), each subgroup was treated as an independent dataset to avoid unit-of-analysis errors and to allow subgroup analyses. If required information was missing or unclear, corresponding authors were contacted to obtain additional data. Studies were excluded from quantitative synthesis only when essential data could not be obtained.

### 4.5. Quality Assessment

Methodological quality was evaluated using the Joanna Briggs Institute (JBI) critical appraisal checklists (https://jbi.global/critical-appraisal-tools, accessed 1 March 2026), applied independently by two reviewers; disagreements were resolved by consensus or a third reviewer. Studies were considered high-quality if more than half of the checklist items were rated “yes.” All included studies met this threshold and were deemed to have acceptable risk of bias (Supplementary Table S3).

### 4.6. Statistical Methods

All statistical analyses were performed using Comprehensive Meta-Analysis software (version 3.0; Biostat, Englewood, NJ, USA). Pooled effect sizes were estimated as standardized mean differences (SMDs) with corresponding 95% confidence intervals (CIs) to compare lipid parameters, including serum Apo A1, HDL-C, LDL-C, total cholesterol (TC), and triglycerides (TGs), between *APOA1* rs670 A allele carriers (GA + AA genotypes) and GG homozygotes. A dominant genetic model (GA + AA vs. GG) was adopted because it was the most consistently reported across the included studies, thereby maximizing the number of eligible datasets and facilitating robust pooled analyses.

Between-study heterogeneity was evaluated using Cochran’s Q test and the I^2^ statistic. An I^2^ value < 50% was considered indicative of low-to-moderate heterogeneity, in which case a fixed-effect model was applied; otherwise, a random-effects model was used. Subgroup analyses were conducted to investigate potential sources of heterogeneity, with particular emphasis on the modifying effect of metabolic status (MetS vs. non-MetS).

Publication bias was assessed through visual inspection of funnel plots and statistically evaluated using Begg’s rank correlation test. Sensitivity analyses were performed using a leave-one-out approach, sequentially removing individual studies to examine the robustness of the pooled estimates. A two-sided *p* < 0.05 was considered statistically significant.

## 5. Conclusions

This meta-analysis demonstrates that the *APOA1* rs670 A allele is associated with higher serum Apo A1 and HDL-C levels, with these effects apparently amplified in individuals with metabolic disorders. Specifically, the magnitude of the association was approximately fivefold greater for Apo A1 and threefold greater for HDL-C in metabolically disordered populations compared with metabolically healthy individuals. These findings support a context-dependent role of rs670 in regulating Apo A1 and HDL-C levels and suggest that metabolic status may be an important modifier of this genetic effect. Given the substantial heterogeneity between studies and the exploratory nature of the subgroup analyses, these observations warrant confirmation in large, prospective cohort studies with standardized metabolic phenotyping and systematic evaluation of interactions between genotype and environmental factors.

## Data Availability

The data extracted from the included studies are available in the Supplementary Materials. Additional deidentified data are available from the corresponding authors upon reasonable request.

## Supplementary Materials

The following supporting information can be downloaded at: https://www.mdpi.com/article/10.3390/ijms27177622/s1. References [11,12,13,15,16,19,20,21,22,23,24,25,26,27,28,29,30,31,32,33,34,35,36,37,38,39,40,41,42,43,44,45,46,47,48,49,50,51,52,53] are cited in the Supplementary Materials.

## Abbreviations

Apo A1, apolipoprotein A1; HDL-C, high-density lipoprotein cholesterol; JBI, Joanna Briggs Institute; LDL-C, low-density lipoprotein cholesterol; MetS, metabolic syndrome; PRISMA, Preferred Reporting Items for Systematic Reviews and Meta-Analyses; SMD, standardized mean difference; TC, total cholesterol; TGs, triglycerides.
