# Supplementary material for: Context-Dependent Associations of the APOA1 rs670 Polymorphism with Apolipoprotein A1 and HDL Cholesterol: A Systematic Review and Meta-Analysis
Source: Int J Mol Sci. 2026 Aug 25;27(17):7622. doi: 10.3390/ijms27177622 (PMC13566318; doi:10.3390/ijms27177622)
Supplement: Supplementary file 1 [file ijms-27-07622-s001.zip › ijms-4473349-supplementary.pdf]

Supplementary Online Materials

Association of the *APOA1* rs670 Polymorphism with Apolipoprotein A1 and HDL Cholesterol Levels: A Meta-Analysis Stratified by Metabolic Status

Chien-Yi Chan [1,†], Yi-Chen Huang [2,†], Ming-Fen Lee [2], Chun-Yin Huang [2,*], Wen-Chieh Wu [2,*]

* Correspondence: chuang@mail.cmu.edu.tw (C.-Y.H.); wcwu@mail.cmu.edu.tw (W.-C.W.); Tel.: +886-4-2205-3366 (ext. 7515 or 7505) (C.-Y.H. & W.-C.W.)

† Chien-Yi Chan and Yi-Chen Huang contributed equally to this article as first authors.

Supplementary Table S1. PRISMA 2020 checklist of the current meta-analysis.
Supplementary Table S2. Detailed search strategies and results in each database.
Supplementary Table S3. Characteristics of the included studies.
Supplementary Figure S1. (**a–e**) Sensitivity analysis of serum APOA1, HDL-C, LDL-C, TC, and TG SMDs between APOA1 rs670 genotypes.
Supplementary Figure S2. (**a–c**) Forest plot of LDL-C, TC, and TG SMDs between *APOA1* rs670 genotypes.
Supplementary Figure S3. (**a–c**) Forest plot of LDL-C, TC, and TG SMDs between *APOA1* rs670 genotypes stratified by metabolic status (MetS vs non-MetS).
Supplementary Figure S4. (**a–e**) Funnel plots for publication bias of serum APOA1, HDL-C, LDL-C, TC, and TG SMDs between APOA1 rs670 genotypes.

References

Supplementary Table S1. PRISMA 2020 checklist of the current meta-analysis.

Section and Topic	Item #	Checklist item	Location where item is reported
**TITLE**			
Title	1	Identify the report as a systematic review.	Page 1
**ABSTRACT**			
Abstract	2	See the PRISMA 2020 for Abstracts checklist.	Page 2~3
**INTRODUCTION**			
Rationale	3	Describe the rationale for the review in the context of existing knowledge.	Page 4~5
Objectives	4	Provide an explicit statement of the objective(s) or question(s) the review addresses.	Page 4~5
**METHODS**			
Eligibility criteria	5	Specify the inclusion and exclusion criteria for the review and how studies were grouped for the syntheses.	Page 7~8
Information sources	6	Specify all databases, registers, websites, organisations, reference lists and other sources searched or consulted to identify studies. Specify the date when each source was last searched or consulted.	Search strategy, Page 6~7
Search strategy	7	Present the full search strategies for all databases, registers and websites, including any filters and limits used.	Search strategy, Page 6~7 and Supplementary Table S2
Selection process	8	Specify the methods used to decide whether a study met the inclusion criteria of the review, including how many reviewers screened each record and each report retrieved, whether they worked independently, and if applicable, details of automation tools used in the process.	Search strategy, Page 6~7<br>Quality assessment, Page 9
Data collection process	9	Specify the methods used to collect data from reports, including how many reviewers collected data from each report, whether they worked independently, any processes for obtaining or confirming data from study investigators, and if applicable, details of automation tools used in the process.	Data extraction, Page 8~9
Data items	10a	List and define all outcomes for which data were sought. Specify whether all results that were compatible with each outcome domain in each study were sought (e.g. for all measures, time points, analyses), and if not, the methods used to decide which results to collect.	Selection criteria, Page 7~8<br>Data extraction, Page 8~9
	10b	List and define all other variables for which data were sought (e.g. participant and intervention characteristics, funding sources). Describe	Data extraction, Page 8~9,

Section and Topic	Item #	Checklist item	Location where item is reported
		any assumptions made about any missing or unclear information.	Statistical methods, Page 10~11, and Supplementary Table S3
Study risk of bias assessment	11	Specify the methods used to assess risk of bias in the included studies, including details of the tool(s) used, how many reviewers assessed each study and whether they worked independently, and if applicable, details of automation tools used in the process.	Quality assessment, Page 9~10
Effect measures	12	Specify for each outcome the effect measure(s) (e.g. risk ratio, mean difference) used in the synthesis or presentation of results.	Statistical methods, Page 10
Synthesis methods	13a	Describe the processes used to decide which studies were eligible for each synthesis (e.g. tabulating the study intervention characteristics and comparing against the planned groups for each synthesis (item #5)).	Selection criteria, Page 7~8, Data extraction, Page 8~9
	13b	Describe any methods required to prepare the data for presentation or synthesis, such as handling of missing summary statistics, or data conversions.	Statistical methods, Page 10~11
	13c	Describe any methods used to tabulate or visually display results of individual studies and syntheses.	Statistical methods, Page 10~11
	13d	Describe any methods used to synthesize results and provide a rationale for the choice(s). If meta-analysis was performed, describe the model(s), method(s) to identify the presence and extent of statistical heterogeneity, and software package(s) used.	Statistical methods, Page 10~11
	13e	Describe any methods used to explore possible causes of heterogeneity among study results (e.g. subgroup analysis, meta-regression).	Statistical methods, Page 10~11
	13f	Describe any sensitivity analyses conducted to assess robustness of the synthesized results.	Statistical methods, Page 11
Reporting bias assessment	14	Describe any methods used to assess risk of bias due to missing results in a synthesis (arising from reporting biases).	Statistical methods, Page 11
Certainty assessment	15	Describe any methods used to assess certainty (or confidence) in the body of evidence for an outcome.	Quality assessment, Page 9~10
**RESULTS**			
Study selection	16a	Describe the results of the search and selection process, from the number of records identified in the search to the number of studies	Search strategy, Page 6~7,

Section and Topic	Item #	Checklist item	Location where item is reported
		included in the review, ideally using a flow diagram.	characteristics, Page 12, and Figure 1
	16b	Cite studies that might appear to meet the inclusion criteria, but which were excluded, and explain why they were excluded.	Search strategy, Page 6~7 and Figure 1
Study characteristics	17	Cite each included study and present its characteristics.	Study characteristics, Page 12 and Supplementary Table S3
Risk of bias in studies	18	Present assessments of risk of bias for each included study.	Quality assessment, Page 9~10 and Supplementary Table S3
Results of individual studies	19	For all outcomes, present, for each study: (a) summary statistics for each group (where appropriate) and (b) an effect estimate and its precision (e.g. confidence/credible interval), ideally using structured tables or plots.	Results, Page 12~16 and Figure 2~5
Results of syntheses	20a	For each synthesis, briefly summarise the characteristics and risk of bias among contributing studies.	Study characteristics, Page 12 and Supplementary Table S3
	20b	Present results of all statistical syntheses conducted. If meta-analysis was done, present for each the summary estimate and its precision (e.g. confidence/credible interval) and measures of statistical heterogeneity. If comparing groups, describe the direction of the effect.	Results, Page 12~16, Figure 2~5, and Supplementary Fig. S2a-c
	20c	Present results of all investigations of possible causes of heterogeneity among study results.	Results, Page 12~16, Figure 2~5, and Supplementary Fig. S2a-c
	20d	Present results of all sensitivity analyses conducted to assess the robustness of the synthesized results.	Results, Page 13~15 and Supplementary Fig. S1a-e
Reporting biases	21	Present assessments of risk of bias due to missing results (arising from reporting biases) for each synthesis assessed.	Publication bias, Page 15~16 and Supplementary Fig. S4a-e
Certainty of	22	Present assessments of certainty (or confidence) in the body of evidence for each outcome assessed.	Quality assessment, Page

Section and Topic	Item #	Checklist item	Location where item is reported
evidence			9~10 and Discussion, Page 21~22
**DISCUSSION**			
Discussion	23a	Provide a general interpretation of the results in the context of other evidence.	Discussion, Page 17~21
	23b	Discuss any limitations of the evidence included in the review.	Discussion, Page 22
	23c	Discuss any limitations of the review processes used.	Discussion, Page 22
	23d	Discuss implications of the results for practice, policy, and future research.	Discussion, Page 22~23 and Conclusion, Page 23
**OTHER INFORMATION**			
Registration and protocol	24a	Provide registration information for the review, including register name and registration number, or state that the review was not registered.	General Guidelines, Page 6
	24b	Indicate where the review protocol can be accessed, or state that a protocol was not prepared.	General Guidelines, Page 6 and Supplementary Table S1
	24c	Describe and explain any amendments to information provided at registration or in the protocol.	General Guidelines, Page 6
Support	25	Describe sources of financial or non-financial support for the review, and the role of the funders or sponsors in the review.	Funding, Page 24
Competing interests	26	Declare any competing interests of review authors.	Conflicts of Interest, Page 1
Availability of data, code and other materials	27	Report which of the following are publicly available and where they can be found: template data collection forms; data extracted from included studies; data used for all analyses; analytic code; any other materials used in the review.	Data extraction, Page 8~9 and Supplementary Table S1~S3

Supplementary Table S2. Detailed search strategies and results in each database.

Database	Keyword	Date	Result
PubMed	("Apolipoprotein A1"[MeSH] OR APOA1 OR "apolipoprotein A-I") AND ("genetic variation"[MeSH] OR polymorphism OR SNP OR rs670 OR "-75 G>A") AND ("metabolic syndrome"[MeSH] OR hypertriglyceridemia OR HDL-C OR "central obesity" OR hyperglycemia OR hypertension)	2026/3/10	1120
Embase	(Apolipoprotein A1 OR APOA1 OR "apolipoprotein A-I") AND (genetic variation OR polymorphism OR SNP OR rs670 OR "-75 G>A") AND (metabolic syndrome OR hypertriglyceridemia OR HDL-C OR "central obesity" OR hyperglycemia OR hypertension)	2026/3/10	920
Web of Science	(Apolipoprotein A1 OR APOA1 OR "apolipoprotein A-I") AND (genetic variation OR polymorphism OR SNP OR rs670 OR "-75 G>A") AND (metabolic syndrome OR hypertriglyceridemia OR HDL-C OR "central obesity" OR hyperglycemia OR hypertension)	2026/3/10	41
Cochrane Library	(Apolipoprotein A1 OR APOA1 OR "apolipoprotein A-I") AND (genetic variation OR polymorphism OR SNP OR rs670 OR "-75 G>A") AND (metabolic syndrome OR hypertriglyceridemia OR HDL-C OR "central obesity" OR hyperglycemia OR hypertension)	2026/3/10	468
ClinicalTrials.gov	(Apolipoprotein A1 OR APOA1 OR "apolipoprotein A-I") AND (genetic variation OR polymorphism OR SNP OR rs670 OR "-75 G>A") AND (metabolic syndrome OR hypertriglyceridemia OR HDL-C OR "central obesity" OR hyperglycemia OR hypertension)	2026/3/10	1

Supplementary Table S3. Characteristics of the included studies.

Study (ref)	Study design	Country	Study participants	Sample size, n	Age	Genotyping method	Quality score
**Chhabra, 2005 [1]**	cross sectional study	India	Northern Indians with or without CAD	200	53.07 ± 10.37 (with CAD) and 51.14 ± 9.70 (without CAD)	PCR-RFLP	7/8
**Daneshpour, 2012 [2]**	cross-sectional study	Iran	Tehranian adults	823	44.4 ± 15.8	PCR-RFLP	8/8
**Bora, 2017 [3]**	case-control study	India	Natives of Assam with or without decreased HDL-C	200	43.12 ± 11.64 (cases) and 42.95 ± 11.60 (controls)	PCR-RFLP	8/10
**Sigurdsson, 1992 [4]**	cross-sectional study	Iceland	Icelandic men and women	315	41.1 ± 1.2 (men) and 42.6 ± 1.2 (women)	PCR-RFLP	8/8
**de Franca, 2005 [5]**	cross-sectional study	Brazil	Healthy Brazilian children of mixed ethnicity	414	8.9 ± 2.9	PCR-RFLP	8/8
**Rai, 2008 [6]**	case-control study	India	Patients with CAD and healthy controls	240	41.51 ± 8.84 (cases) and 37.25 ± 6.18 (controls)	PCR-RFLP	7/10
**Larson, 2002 [7]**	cross-sectional study	USA	Caucasian men and women	734	56.1 (females) and 58.7 (males)	PCR-RFLP	8/8
**Li, 2008 [8]**	cross-sectional study	China	Healthy Hei Yi Zhuang and Han Chinese	1038	42.47 ± 17.75 (Hei Yi Zhuang) and 42.03 ± 17.21 (Han)	PCR-RFLP	7/8

**Hsu, 2019 [9]**	case-control study	Taiwan	Han Chinese with or without stroke	1751	63.18 ± 11.49	PCR & MALDI-TOF MS	8/10
**Wang, 2018 [10]**	case-control study	China	Uyghur and Kazakh adults with or without dyslipidemia	1486	47.16 ± 11.45 (cases) and 41.52 ± 13.56 (controls)	TaqMan assay (real-time qPCR)	7/10
**Ding, 2012 [11]**	case-control study	China	Han Chinese with or without ACS	483	59 (cases) and 58 (controls)	PCR-RFLP	7/10
**Al-Bustan, 2013 [12]**	cross-sectional study	Kuwait	Healthy Kuwaiti volunteers	549	29 ± 0.54	PCR & Sequencing	8/8
**Dallinga-Thie, 1996 [13]**	cohort study	The Netherlands	FCH probands, relatives, and spouses	585	53 ± 14 (probands), 39 ± 16 (relatives), 48 ± 16 (spouses)	PCR-RFLP	6/11
**Xiao, 2008 [14]**	case-control study	Australia	Australian men and women with or without CAD	1543	50 ± 5 (cases) and 52 ± 13 (controls)	PCR-RFLP	6/10
**Chen, 2009 [15]**	Cohort study	Brazil	Brazilian elderly	304	Elderly(≥60 years)	PCR-RFLP	6/11
**Talmud, 1994 [16]**	Case-control study	Europe (12 countries)	Healthy offspring of fathers with MI and controls	1643	18-26	PCR & Oligonucleotide melting	9/10

**Bai, 1996 [17]**	case-control study	Japan	Japanese with or without CAD	416	64 ± 12 (CAD) and 59 ± 12 (controls)	PCR-RFLP & Sequencing	6/10
**Zhu, 2001 [18]**	case-control study	China	Han Chinese with or without HTG	389	54.7 ± 12.6 (HTG) and 51.7 ± 10.9 (controls)	PCR-RFLP	5/10
**Song, 2014 [19]**	cross-sectional study	China	Healthy adolescents with different BMI	723	16.86 ± 0.59	PCR-RFLP	7/8
**Ordovas, 2002 [20]**	cross-sectional study	USA	Men and women	1577	55.0 ± 9.4 (men) and 54.3 ± 9.5 (women)	PCR	8/8
**Jia, 2005 [21]**	cross-sectional study	China	Chinese adults	307	56.7 ± 11.2 (men) and 56.9 ± 11.6 (women)	PCR-RFLP	5/8
**Reguero, 1998 [22]**	case-control study	Spain	Patients with early coronary disease and controls	176	43 ± 5 (cases)	PCR-RFLP	6/10
**Costa, 2024 [23]**	Cross-sectional study	Brazil	COVID-19 outpatients and controls	167	39.7 ± 13.9	PCR-RFLP	8/8
**Phillips, 2011 [24]**	case-control study	France	Subjects with or without MetS	1754	35–60 (women) and 45–60 (men)	PCR-RFLP	8/10
**Izaola, 2020 [25]**	randomized clinical trial	Spain	Obese human subjects	268	49.3 ± 9.1	PCR	7/13

**Padmaja, 2009 [26]**	cross-sectional study	India	Tamilians	185	48.6 ± 6.9	PCR-RFLP	7/8
**Ramezani-Jolfaie, 2020 [27]**	randomized clinical trial	Iran	Adults with and without T2DM	168	> 18	PCR-RFLP	7/13
**de Luis, 2019-c [28]**	randomized clinical trial	Spain	Obese subjects	360	25–65	PCR	3/13
**de Luis, 2019-a [29]**	cross-sectional study	Spain	Adult obese subjects	1000	46.3 ± 9.1	PCR	7/8
**de Luis, 2018-a [30]**	Quasi-experimental study	Spain	Obese subjects	82	20-65	PCR	5/9
**Wang, 1996 [31]**	cross-sectional study	Australia	Healthy Caucasians	243	28.3 ± 17.9	PCR-RFLP	6/8
**Jeenah, 1990 [32]**	case-control study	UK	Healthy men	94	45-55	PCR-RFLP	6/10
**Supajaree, 2022 [33]**	cross-sectional study	Thailand	Thai elderly	355	67.65 ± 4.11	PCR	7/8
**de Luis, 2019-b [34]**	randomized clinical trial	Spain	Obese human subjects	282	49.7 ± 9.2	PCR	6/13
**de Luis, 2018-b [35]**	cohort study	Spain	Morbid obese patients	63	47.3 ± 8.1	PCR	6/11

**Zou, 2003 [36]**	case-control study	China	Chinese with or without CAD	137	54 (CAD) and 52 (controls)	PCR-RFLP	8/10
**Dawar, 2010 [37]**	case-control study	India	Patients with MI and controls	100	> 40	PCR-RFLP	5/10
**Bora, 2016 [38]**	cross-sectional study	India	Native population of Assam	150	42.87 ± 11.86	PCR-RFLP	5/8
**Swanson, 2016 [39]**	case-control study	USA	Patients with Parkinson's disease and normal controls	539	65-67 (median)	PCR-RFLP	6/10
**Chien, 2008 [40]**	case-control study	Taiwan	Taiwanese with or without hypertriglyceridemia	589	55.6 (cases) and 50.3 (controls)	PCR-RFLP	7/10

Study name	Statistics with study removed				
	Point	Standard error	Lower limit	Upper limit	p-Value
Chhabra, 2005	0.215	0.052	0.113	0.317	0.000
Bai, 1996b	0.211	0.053	0.106	0.315	0.000
Sigurdsson, 1992b	0.206	0.053	0.102	0.309	0.000
Rai, 2008b	0.205	0.053	0.100	0.309	0.000
Bora, 2016	0.206	0.054	0.101	0.310	0.000
Larson, 2002a	0.207	0.054	0.101	0.313	0.000
Ramezani-Jolfaie, 2020	0.202	0.053	0.098	0.306	0.000
Daneshpour, 2012	0.208	0.054	0.101	0.314	0.000
Jia, 2005	0.206	0.054	0.100	0.312	0.000
Talmud, 1994b	0.207	0.055	0.099	0.315	0.000
Dallinga-Thie, 1996	0.206	0.054	0.099	0.313	0.000
Li, 2008	0.206	0.055	0.099	0.314	0.000
Ordovas, 2002b	0.206	0.055	0.097	0.314	0.000
Ordovas, 2002a	0.205	0.055	0.097	0.314	0.000
Larson, 2002b	0.203	0.054	0.097	0.310	0.000
Talmud, 1994a	0.203	0.055	0.095	0.312	0.000
Phillips, 2011	0.206	0.057	0.093	0.318	0.000
Costa, 2024	0.198	0.053	0.093	0.302	0.000
Reguero, 1998	0.198	0.054	0.092	0.303	0.000
Bai, 1996a	0.196	0.054	0.091	0.301	0.000
Swanson, 2016a	0.195	0.054	0.089	0.301	0.000
Rai, 2008a	0.194	0.053	0.089	0.299	0.000
Swanson, 2016b	0.191	0.053	0.087	0.296	0.000
Jeenah, 1990	0.187	0.052	0.084	0.290	0.000
Sigurdsson, 1992a	0.184	0.052	0.082	0.287	0.000
Zou, 2003	0.176	0.051	0.076	0.277	0.001
Zhu, 2001	0.152	0.045	0.063	0.240	0.001
Dawar, 2010	0.149	0.044	0.063	0.236	0.001
	0.197	0.052	0.095	0.299	0.000

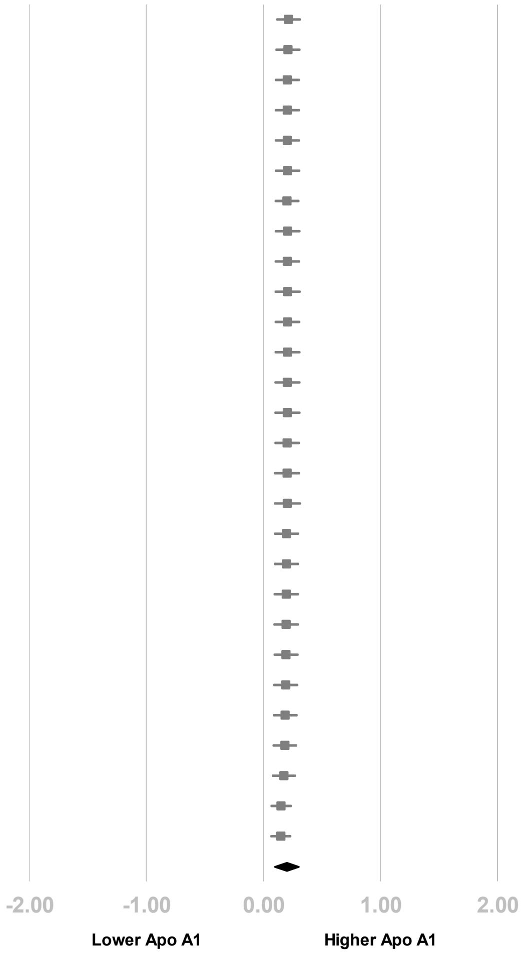

Supplementary Figure S1. (**a**) Sensitivity analysis of serum APOA1 SMDs between APOA1 rs670 genotypes.

Study name	Statistics with study removed				
	Point	Standard error	Lower limit	Upper limit	p-Value
Chhabra, 2005	0.148	0.036	0.077	0.220	0.000
Daneshpour, 2012	0.148	0.036	0.076	0.219	0.000
de Franca, 2005b	0.146	0.037	0.074	0.218	0.000
Bora, 2017a	0.144	0.037	0.072	0.216	0.000
Sigurdsson, 1992b	0.143	0.037	0.071	0.215	0.000
Rai, 2008a	0.144	0.037	0.071	0.216	0.000
Larson, 2002a	0.145	0.037	0.072	0.217	0.000
Li, 2008	0.145	0.037	0.073	0.218	0.000
Hsu, 2019	0.146	0.037	0.073	0.219	0.000
Wang, 2018b	0.145	0.037	0.072	0.218	0.000
Ding, 2012	0.144	0.037	0.072	0.217	0.000
Al-Bustan, 2013	0.144	0.037	0.072	0.217	0.000
Dallinga-Thie, 1996	0.144	0.037	0.071	0.216	0.000
Larson, 2002b	0.143	0.037	0.070	0.216	0.000
de Franca, 2005c	0.143	0.037	0.070	0.216	0.000
Xiao, 2008b	0.143	0.037	0.070	0.216	0.000
Bora, 2017c	0.142	0.037	0.069	0.215	0.000
Chen, 2009	0.142	0.037	0.069	0.215	0.000
Talmud, 1994b	0.143	0.037	0.069	0.216	0.000
Bai, 1996b	0.142	0.037	0.069	0.214	0.000
Zhu, 2001	0.141	0.037	0.069	0.213	0.000
Song, 2014a	0.142	0.037	0.069	0.214	0.000
Ordovas, 2002a	0.143	0.037	0.069	0.216	0.000
Wang, 2018a	0.142	0.037	0.069	0.216	0.000
Jia, 2005	0.142	0.037	0.069	0.215	0.000
Ordovas, 2002b	0.142	0.038	0.069	0.216	0.000
Song, 2014d	0.141	0.037	0.069	0.214	0.000
Xiao, 2008a	0.142	0.038	0.068	0.216	0.000
Talmud, 1994a	0.141	0.038	0.067	0.214	0.000
Reguero, 1998	0.140	0.037	0.068	0.213	0.000
Bora, 2017b	0.140	0.037	0.067	0.212	0.000
Costa, 2024	0.139	0.037	0.067	0.211	0.000
de Franca, 2005a	0.139	0.037	0.067	0.212	0.000
Phillips, 2011	0.140	0.038	0.066	0.215	0.000
Song, 2014c	0.139	0.037	0.066	0.211	0.000
Izaola, 2020	0.139	0.037	0.067	0.211	0.000
Song, 2014b	0.139	0.037	0.066	0.211	0.000
Bai, 1996a	0.138	0.037	0.065	0.210	0.000
Padmaja, 2009	0.137	0.037	0.065	0.210	0.000
Ramezani-Jolfaie, 2020	0.138	0.037	0.066	0.210	0.000
Rai, 2008b	0.137	0.037	0.065	0.209	0.000
de Luis, 2019-3a	0.136	0.037	0.064	0.208	0.000
de Luis, 2018-1	0.136	0.037	0.064	0.208	0.000
de Luis, 2019-1b	0.135	0.037	0.063	0.207	0.000
Wang, 1996	0.134	0.037	0.062	0.206	0.000
Sigurdsson, 1992a	0.135	0.037	0.063	0.206	0.000
de Luis, 2019-1a	0.133	0.037	0.061	0.205	0.000
Jeenah, 1990	0.134	0.037	0.063	0.206	0.000
de Luis, 2019-3b	0.130	0.036	0.059	0.201	0.000
Supajaree, 2022	0.126	0.035	0.057	0.195	0.000
de Luis, 2019-2	0.118	0.032	0.054	0.182	0.000
de Luis, 2018-2	0.131	0.036	0.060	0.202	0.000
Zou, 2003	0.126	0.036	0.056	0.196	0.000
Dawar, 2010	0.121	0.035	0.053	0.189	0.000
	0.139	0.036	0.068	0.211	0.000

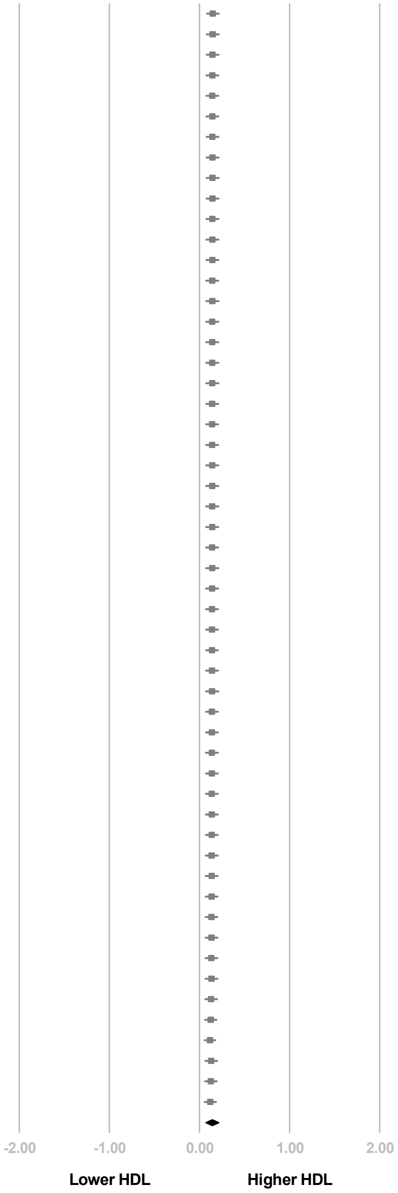

Supplementary Figure S1. (**b**) Sensitivity analysis of serum HDL-C SMDs between APOA1 rs670 genotypes.

Study name	Statistics with study removed				
	Point	Standard error	Lower limit	Upper limit	p-Value
Ramezani-Jolfaie, 2020	0.076	0.037	0.005	0.148	0.037
de Luis, 2018-1	0.077	0.037	0.005	0.148	0.037
de Luis, 2019-1a	0.079	0.036	0.007	0.150	0.031
Bora, 2017b	0.075	0.037	0.003	0.147	0.042
Rai, 2008b	0.075	0.037	0.003	0.147	0.042
Izaola, 2020	0.075	0.037	0.002	0.147	0.043
Chen, 2009	0.076	0.037	0.003	0.148	0.041
Rai, 2008a	0.074	0.037	0.001	0.146	0.047
de Luis, 2019-3b	0.074	0.037	0.001	0.146	0.047
Bai, 1996a	0.074	0.037	0.001	0.147	0.047
Bai, 1996b	0.072	0.037	-0.001	0.145	0.052
Bora, 2017c	0.072	0.037	-0.001	0.145	0.055
Larson, 2002b	0.072	0.037	-0.002	0.145	0.055
Supajaree, 2022	0.072	0.038	-0.002	0.145	0.055
Hsu, 2019	0.072	0.039	-0.004	0.148	0.062
Jia, 2005	0.072	0.037	-0.002	0.145	0.056
Xiao, 2008b	0.072	0.038	-0.002	0.146	0.057
Reguero, 1998	0.071	0.037	-0.002	0.144	0.058
Zhu, 2001	0.070	0.037	-0.002	0.143	0.058
Wang, 2018b	0.071	0.038	-0.003	0.145	0.060
Ordovas, 2002a	0.071	0.038	-0.003	0.145	0.061
de Luis, 2019-2	0.071	0.038	-0.004	0.146	0.062
Song, 2014a	0.070	0.037	-0.003	0.144	0.059
Ordovas, 2002b	0.071	0.038	-0.004	0.145	0.062
Xiao, 2008a	0.070	0.038	-0.005	0.145	0.066
Costa, 2024	0.070	0.037	-0.003	0.142	0.061
de Franca, 2005	0.070	0.038	-0.004	0.144	0.064
Phillips, 2011	0.070	0.039	-0.006	0.146	0.072
Li, 2008	0.069	0.038	-0.005	0.143	0.067
Wang, 2018a	0.069	0.038	-0.005	0.143	0.068
Song, 2014c	0.069	0.037	-0.005	0.142	0.066
Zou, 2003	0.068	0.037	-0.004	0.141	0.065
Song, 2014d	0.068	0.037	-0.005	0.141	0.068
Song, 2014b	0.068	0.037	-0.006	0.141	0.070
Dallinga-Thie, 1996	0.066	0.038	-0.007	0.140	0.077
Padmaja, 2009	0.066	0.037	-0.007	0.139	0.074
Al-Bustan, 2013	0.066	0.038	-0.008	0.139	0.081
Larson, 2002a	0.065	0.037	-0.008	0.138	0.080
Bora, 2017a	0.063	0.037	-0.009	0.135	0.087
Daneshpour, 2012	0.051	0.033	-0.014	0.117	0.123
de Luis, 2018-2	0.063	0.037	-0.009	0.134	0.087
de Luis, 2019-3a	0.048	0.033	-0.016	0.112	0.141
de Luis, 2019-1b	0.050	0.033	-0.016	0.115	0.136
	0.069	0.037	-0.003	0.141	0.059

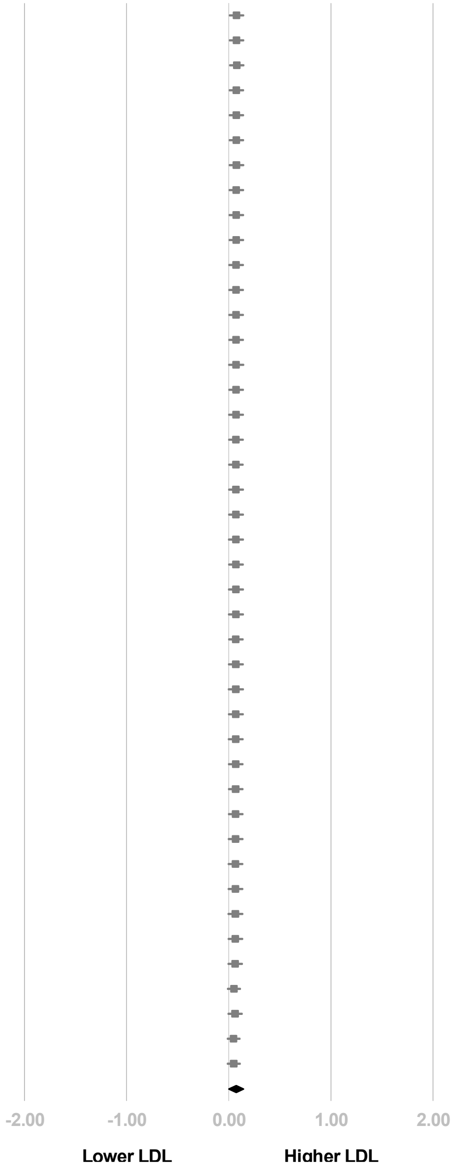

Supplementary Figure S1. (**c**) Sensitivity analysis of serum LDL-C SMDs between APOA1 rs670 genotypes.

Study name	Statistics with study removed				
	Point	Standard error	Lower limit	Upper limit	p-Value
de Luis, 2018-1	0.097	0.032	0.033	0.161	0.003
Ramezani-Jolfaie, 2020	0.089	0.035	0.020	0.157	0.011
Bora, 2017b	0.087	0.035	0.018	0.156	0.013
Rai, 2008b	0.087	0.035	0.017	0.156	0.014
Chen, 2009	0.089	0.035	0.020	0.158	0.012
Rai, 2008a	0.087	0.035	0.017	0.156	0.014
Chhabra, 2005	0.087	0.035	0.018	0.156	0.014
Bai, 1996a	0.087	0.035	0.018	0.156	0.014
de Luis, 2018-2	0.083	0.035	0.014	0.152	0.018
Bai, 1996b	0.085	0.036	0.016	0.155	0.016
Hsu, 2019	0.087	0.036	0.016	0.158	0.017
Larson, 2002b	0.085	0.036	0.015	0.155	0.018
Bora, 2017c	0.084	0.036	0.014	0.154	0.019
Reguero, 1998	0.083	0.036	0.013	0.153	0.019
Costa, 2024	0.083	0.035	0.013	0.152	0.019
Daneshpour, 2012	0.083	0.036	0.013	0.154	0.021
Jia, 2005	0.083	0.036	0.013	0.154	0.020
Phillips, 2011	0.082	0.037	0.010	0.155	0.027
Izaola, 2020	0.082	0.035	0.013	0.152	0.020
Song, 2014a	0.082	0.036	0.012	0.152	0.021
Supajaree, 2022	0.082	0.036	0.011	0.152	0.023
Li, 2008	0.081	0.036	0.010	0.152	0.025
de Luis, 2019-1a	0.081	0.036	0.012	0.151	0.022
Song, 2014c	0.081	0.036	0.011	0.151	0.023
de Franca, 2005	0.081	0.036	0.010	0.151	0.025
de Luis, 2019-2	0.079	0.036	0.008	0.151	0.029
Zhu, 2001	0.081	0.035	0.011	0.150	0.023
Song, 2014d	0.080	0.036	0.010	0.150	0.025
Padmaja, 2009	0.080	0.036	0.010	0.150	0.025
de Luis, 2019-3b	0.080	0.036	0.010	0.150	0.025
Wang, 2018a	0.079	0.036	0.008	0.149	0.029
Al-Bustan, 2013	0.079	0.036	0.008	0.149	0.029
Wang, 2018b	0.078	0.036	0.008	0.149	0.030
Zou, 2003	0.080	0.035	0.011	0.149	0.024
Larson, 2002a	0.078	0.036	0.008	0.148	0.028
Song, 2014b	0.078	0.036	0.009	0.148	0.028
Dallinga-Thie, 1996	0.074	0.035	0.005	0.143	0.035
Bora, 2017a	0.077	0.035	0.008	0.146	0.029
de Luis, 2019-3a	0.065	0.031	0.004	0.125	0.037
de Luis, 2019-1b	0.066	0.031	0.004	0.127	0.036
	0.082	0.035	0.013	0.150	0.019

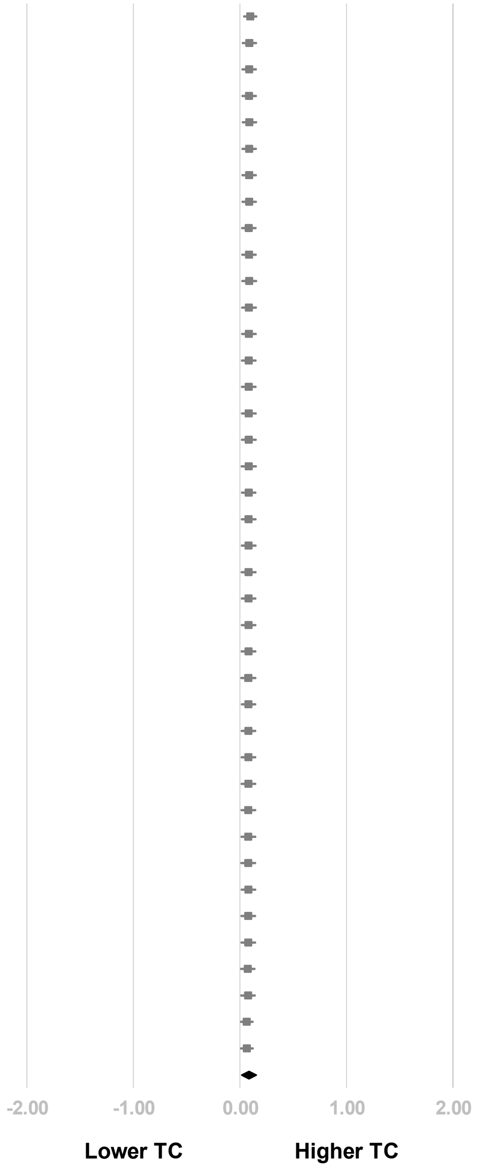

Supplementary Figure S1. (**d**) Sensitivity analysis of serum TC SMDs between APOA1 rs670 genotypes.

Study name	Statistics with study removed				
	Point	Standard error	Lower limit	Upper limit	p-Value
Ramezani-Jolfaie, 2020	-0.013	0.034	-0.078	0.053	0.709
Reguero, 1998	-0.017	0.036	-0.088	0.054	0.636
Supajaree, 2022	-0.014	0.035	-0.082	0.054	0.685
de Luis, 2019-1a	-0.023	0.037	-0.096	0.050	0.530
Ding, 2012	-0.024	0.037	-0.096	0.049	0.524
Izaola, 2020	-0.031	0.038	-0.106	0.043	0.412
de Luis, 2019-1b	-0.032	0.038	-0.107	0.043	0.406
Song, 2014c	-0.032	0.038	-0.107	0.043	0.402
Chien, 2008	-0.033	0.038	-0.108	0.043	0.397
Zou, 2003	-0.034	0.038	-0.109	0.041	0.378
Chen, 2009	-0.035	0.038	-0.110	0.041	0.370
Bai, 1996a	-0.035	0.038	-0.110	0.041	0.368
Al-Bustan, 2013	-0.035	0.039	-0.111	0.041	0.363
Padmaja, 2009	-0.035	0.038	-0.111	0.040	0.358
Costa, 2024	-0.035	0.038	-0.110	0.040	0.356
Bai, 1996b	-0.036	0.038	-0.111	0.040	0.353
Song, 2014b	-0.036	0.038	-0.111	0.040	0.352
Ordovas, 2002a	-0.036	0.039	-0.113	0.040	0.351
de Luis, 2019-2	-0.037	0.039	-0.114	0.040	0.348
Ordovas, 2002b	-0.037	0.039	-0.114	0.039	0.341
Larson, 2002b	-0.037	0.039	-0.112	0.039	0.342
Phillips, 2011	-0.038	0.040	-0.116	0.040	0.339
Bora, 2017c	-0.037	0.038	-0.112	0.039	0.342
Daneshpour, 2012	-0.037	0.039	-0.113	0.039	0.338
Hsu, 2019	-0.039	0.040	-0.117	0.040	0.336
Bora, 2017b	-0.036	0.038	-0.111	0.039	0.342
Xiao, 2008a	-0.038	0.039	-0.115	0.039	0.332
de Franca, 2005c	-0.038	0.039	-0.114	0.038	0.330
Song, 2014d	-0.037	0.038	-0.112	0.038	0.333
de Luis, 2018-1	-0.036	0.038	-0.111	0.038	0.339
Chhabra, 2005	-0.037	0.038	-0.112	0.038	0.333
Rai, 2008a	-0.037	0.038	-0.112	0.038	0.335
Song, 2014a	-0.038	0.038	-0.114	0.037	0.319
Bora, 2017a	-0.038	0.038	-0.113	0.037	0.323
Wang, 2018a	-0.040	0.039	-0.116	0.036	0.303
Zhu, 2001	-0.038	0.038	-0.113	0.037	0.319
Xiao, 2008b	-0.041	0.039	-0.117	0.034	0.286
Rai, 2008b	-0.039	0.038	-0.114	0.036	0.305
Wang, 2018b	-0.042	0.038	-0.117	0.033	0.271
Larson, 2002a	-0.041	0.038	-0.117	0.034	0.279
Li, 2008	-0.043	0.038	-0.118	0.033	0.267
Dallinga-Thie, 1996	-0.042	0.038	-0.117	0.033	0.267
Jia, 2005	-0.047	0.037	-0.120	0.025	0.199
de Luis, 2018-2	-0.042	0.038	-0.116	0.032	0.269
	-0.035	0.038	-0.109	0.039	0.352

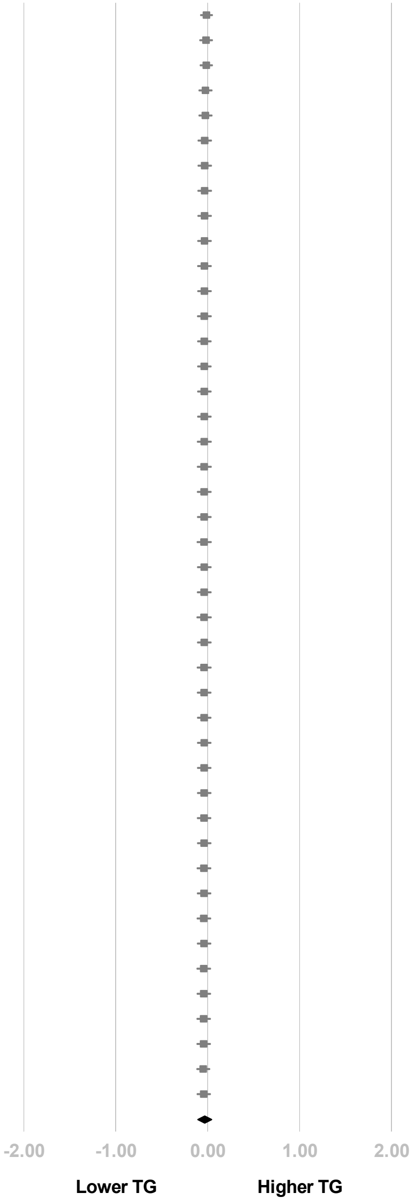

Supplementary Figure S1. (**e**) Sensitivity analysis of serum TG SMDs between APOA1 rs670 genotypes.

Study name	Statistics for each study						Weight (
	Std diff in means	Standard error	Lower limit	Upper limit	Total	p-Value	Relative weight
Ramezani-Jolfaie, 2020	-0.526	0.275	-1.065	0.013	73	0.056	1.18
de Luis, 2018-1	-0.435	0.237	-0.900	0.031	82	0.067	1.42
de Luis, 2019-1a	-0.427	0.180	-0.779	-0.075	134	0.018	1.91
Bora, 2017b	-0.277	0.207	-0.683	0.128	100	0.180	1.66
Rai, 2008b	-0.263	0.201	-0.657	0.130	100	0.190	1.71
Izaola, 2020	-0.244	0.204	-0.644	0.156	131	0.232	1.69
Chen, 2009	-0.194	0.138	-0.465	0.077	214	0.160	2.35
Rai, 2008a	-0.153	0.181	-0.508	0.202	140	0.399	1.90
de Luis, 2019-3b	-0.140	0.164	-0.462	0.181	174	0.392	2.07
Bai, 1996a	-0.134	0.158	-0.444	0.175	211	0.395	2.14
Bai, 1996b	-0.063	0.150	-0.357	0.231	205	0.675	2.22
Bora, 2017c	-0.036	0.145	-0.321	0.248	200	0.802	2.28
Larson, 2002b	-0.027	0.114	-0.251	0.196	372	0.810	2.64
Supajaree, 2022	-0.025	0.106	-0.233	0.183	355	0.812	2.73
Hsu, 2019	-0.021	0.048	-0.115	0.073	1751	0.657	3.34
Jia, 2005	-0.018	0.115	-0.244	0.208	307	0.875	2.62
Xiao, 2008b	-0.013	0.090	-0.190	0.164	554	0.885	2.91
Reguero, 1998	0.000	0.160	-0.313	0.313	176	1.000	2.12
Zhu, 2001	0.001	0.214	-0.419	0.420	199	0.998	1.60
Wang, 2018b	0.008	0.083	-0.155	0.172	690	0.920	2.99
Ordovas, 2002a	0.013	0.080	-0.143	0.169	755	0.874	3.03
de Luis, 2019-2	0.016	0.071	-0.123	0.154	902	0.827	3.12
Song, 2014a	0.019	0.149	-0.274	0.311	180	0.901	2.23
Ordovas, 2002b	0.022	0.077	-0.128	0.172	822	0.773	3.06
Xiao, 2008a	0.034	0.064	-0.092	0.159	1106	0.600	3.19
Costa, 2024	0.046	0.226	-0.398	0.489	81	0.840	1.51
de Franca, 2005	0.046	0.094	-0.139	0.231	449	0.628	2.86
Phillips, 2011	0.052	0.052	-0.050	0.154	1754	0.318	3.30
Li, 2008	0.070	0.085	-0.097	0.236	564	0.414	2.97
Wang, 2018a	0.080	0.086	-0.089	0.248	743	0.355	2.96
Song, 2014c	0.101	0.149	-0.192	0.393	181	0.499	2.23
Zou, 2003	0.115	0.200	-0.277	0.507	137	0.565	1.72
Song, 2014d	0.120	0.150	-0.173	0.413	180	0.423	2.23
Song, 2014b	0.144	0.150	-0.149	0.437	182	0.336	2.23
Dallinga-Thie, 1996	0.167	0.099	-0.027	0.362	408	0.092	2.81
Padmaja, 2009	0.194	0.152	-0.103	0.491	185	0.201	2.20
Al-Bustan, 2013	0.196	0.091	0.017	0.375	529	0.032	2.90
Larson, 2002a	0.224	0.121	-0.013	0.460	359	0.064	2.56
Bora, 2017a	0.442	0.207	0.038	0.847	100	0.032	1.67
Daneshpour, 2012	0.665	0.097	0.476	0.855	586	0.000	2.84
de Luis, 2018-2	0.672	0.290	0.104	1.241	63	0.020	1.10
de Luis, 2019-3a	1.183	0.169	0.851	1.514	186	0.000	2.02
de Luis, 2019-1b	1.243	0.194	0.863	1.623	148	0.000	1.78
	0.069	0.037	-0.003	0.141	16768	0.059	

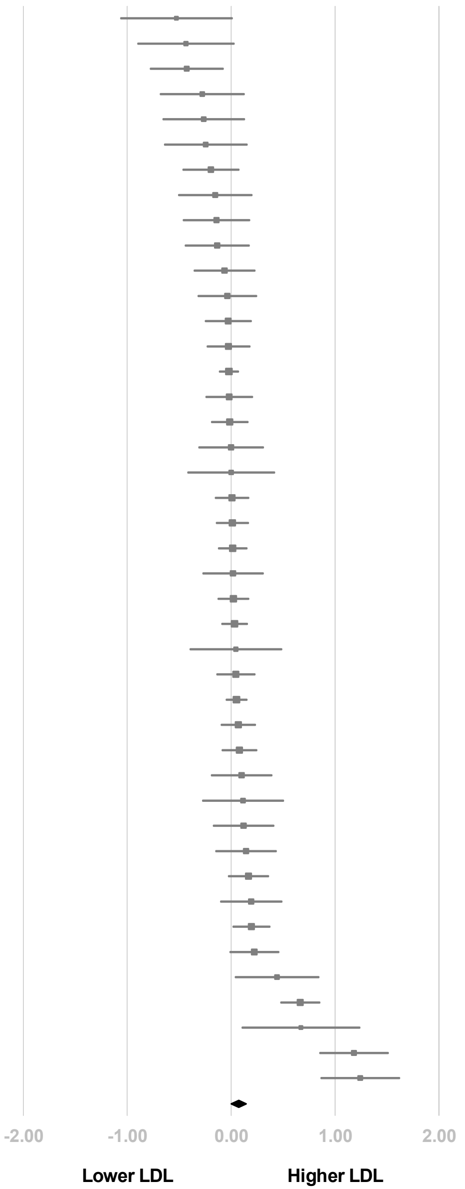

Supplementary Figure S2. (**a**) Forest plot of LDL-C SMDs between *APOA1* rs670 genotypes

Study name	Std diff in means	Standard error	Lower limit	Upper limit	Total	p-Value	Relative weight
de Luis, 2018-1	-1.028	0.248	-1.515	-0.541	82	0.000	1.36
Ramezani-Jolfaie, 2020	-0.477	0.274	-1.015	0.061	73	0.082	1.18
Bora, 2017b	-0.216	0.207	-0.621	0.189	100	0.295	1.73
Rai, 2008b	-0.179	0.200	-0.571	0.214	100	0.373	1.79
Chen, 2009	-0.177	0.136	-0.443	0.089	222	0.191	2.64
Rai, 2008a	-0.156	0.181	-0.511	0.199	140	0.390	2.01
Chhabra, 2005	-0.142	0.161	-0.458	0.174	164	0.380	2.27
Bai, 1996a	-0.134	0.158	-0.444	0.175	211	0.395	2.31
de Luis, 2018-2	-0.067	0.284	-0.624	0.489	63	0.813	1.12
Bai, 1996b	-0.063	0.150	-0.357	0.231	205	0.675	2.42
Hsu, 2019	-0.049	0.048	-0.143	0.045	1751	0.308	4.06
Larson, 2002b	-0.027	0.114	-0.251	0.196	372	0.810	2.99
Bora, 2017c	-0.000	0.145	-0.284	0.284	200	0.999	2.50
Reguero, 1998	0.018	0.160	-0.295	0.331	176	0.908	2.29
Costa, 2024	0.019	0.226	-0.425	0.462	81	0.934	1.54
Daneshpour, 2012	0.024	0.095	-0.161	0.210	586	0.797	3.32
Jia, 2005	0.027	0.115	-0.199	0.253	307	0.815	2.97
Phillips, 2011	0.049	0.052	-0.053	0.152	1754	0.345	4.00
Izaola, 2020	0.052	0.203	-0.347	0.450	131	0.800	1.76
Song, 2014a	0.061	0.149	-0.231	0.354	180	0.682	2.44
Supajaree, 2022	0.080	0.106	-0.128	0.288	355	0.450	3.12
Li, 2008	0.081	0.085	-0.085	0.248	564	0.339	3.49
de Luis, 2019-1a	0.092	0.178	-0.257	0.441	134	0.606	2.05
Song, 2014c	0.105	0.149	-0.188	0.397	181	0.482	2.44
de Franca, 2005	0.105	0.105	-0.099	0.310	414	0.313	3.15
de Luis, 2019-2	0.137	0.071	-0.002	0.276	902	0.053	3.72
Zhu, 2001	0.142	0.214	-0.277	0.562	199	0.506	1.66
Song, 2014d	0.144	0.150	-0.149	0.437	180	0.335	2.43
Padmaja, 2009	0.149	0.151	-0.148	0.446	185	0.325	2.41
de Luis, 2019-3b	0.158	0.164	-0.164	0.479	174	0.336	2.23
Wang, 2018a	0.161	0.086	-0.008	0.330	743	0.061	3.47
Al-Bustan, 2013	0.165	0.090	-0.011	0.341	549	0.067	3.41
Wang, 2018b	0.169	0.083	0.005	0.332	690	0.043	3.51
Zou, 2003	0.176	0.200	-0.216	0.568	137	0.378	1.80
Larson, 2002a	0.198	0.121	-0.038	0.435	359	0.100	2.88
Song, 2014b	0.225	0.150	-0.069	0.518	182	0.134	2.43
Dallinga-Thie, 1996	0.311	0.100	0.115	0.506	408	0.002	3.24
Bora, 2017a	0.356	0.206	-0.048	0.759	100	0.084	1.74
de Luis, 2019-3a	0.980	0.166	0.655	1.304	186	0.000	2.21
de Luis, 2019-1b	1.067	0.190	0.695	1.440	148	0.000	1.91
	0.082	0.035	0.013	0.150	13688	0.019	

Statistics for each study; Weight (

Std diff in means and 95% CI

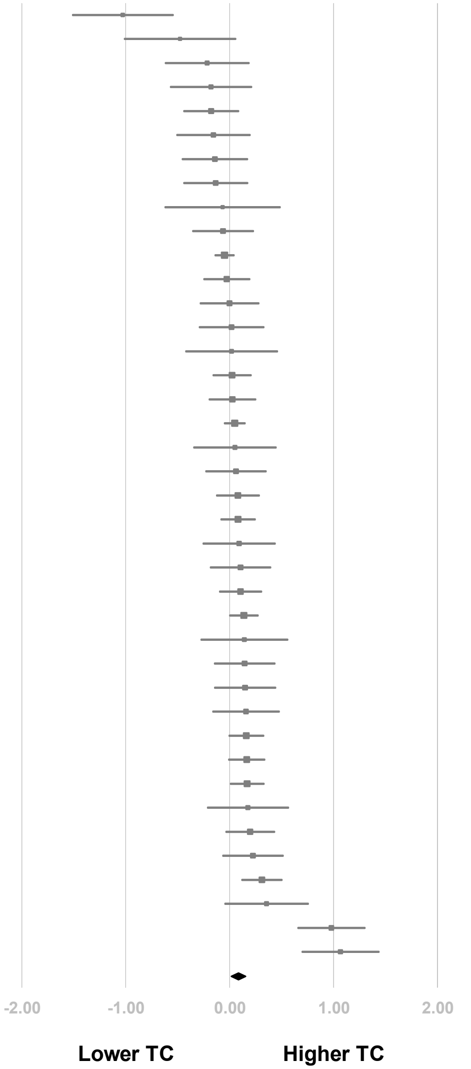

Supplementary Figure S2. (**b**) Forest plot of TC SMDs between *APOA1* rs670 genotypes

Study name	Statistics for each study						Weight (
	Std diff in means	Standard error	Lower limit	Upper limit	Total	p-Value	Relative weight
Ramezani-Jolfaie, 2020	-2.273	0.330	-2.921	-1.626	73	0.000	0.94
Reguero, 1998	-0.826	0.166	-1.151	-0.502	176	0.000	2.02
Supajaree, 2022	-0.683	0.109	-0.897	-0.469	355	0.000	2.59
de Luis, 2019-1a	-0.612	0.182	-0.968	-0.256	134	0.001	1.87
Ding, 2012	-0.396	0.093	-0.579	-0.214	483	0.000	2.76
Izaola, 2020	-0.263	0.204	-0.662	0.137	131	0.198	1.68
de Luis, 2019-1b	-0.216	0.180	-0.569	0.137	148	0.231	1.89
Song, 2014c	-0.177	0.149	-0.470	0.116	181	0.237	2.18
Chien, 2008	-0.148	0.128	-0.399	0.103	245	0.248	2.40
Zou, 2003	-0.127	0.200	-0.519	0.265	137	0.527	1.72
Chen, 2009	-0.072	0.135	-0.337	0.193	222	0.595	2.32
Bai, 1996a	-0.071	0.158	-0.381	0.238	211	0.651	2.10
Al-Bustan, 2013	-0.048	0.090	-0.223	0.128	549	0.595	2.79
Padmaja, 2009	-0.038	0.151	-0.334	0.259	185	0.804	2.16
Costa, 2024	-0.037	0.226	-0.480	0.406	81	0.871	1.51
Bai, 1996b	-0.020	0.150	-0.314	0.275	205	0.897	2.17
Song, 2014b	-0.018	0.149	-0.311	0.275	182	0.904	2.18
Ordovas, 2002a	-0.015	0.080	-0.171	0.141	755	0.853	2.89
de Luis, 2019-2	-0.009	0.071	-0.147	0.130	902	0.904	2.97
Ordovas, 2002b	0.011	0.077	-0.139	0.161	822	0.889	2.91
Larson, 2002b	0.012	0.114	-0.211	0.235	372	0.918	2.54
Phillips, 2011	0.014	0.052	-0.088	0.117	1754	0.782	3.11
Bora, 2017c	0.016	0.145	-0.268	0.300	200	0.910	2.22
Daneshpour, 2012	0.019	0.095	-0.166	0.205	586	0.838	2.74
Hsu, 2019	0.022	0.048	-0.072	0.116	1751	0.650	3.14
Bora, 2017b	0.026	0.206	-0.378	0.430	100	0.901	1.67
Xiao, 2008a	0.032	0.064	-0.094	0.157	1106	0.620	3.02
de Franca, 2005c	0.045	0.104	-0.160	0.249	414	0.670	2.64
Song, 2014d	0.047	0.149	-0.246	0.340	180	0.753	2.18
de Luis, 2018-1	0.048	0.235	-0.413	0.509	82	0.838	1.45
Chhabra, 2005	0.049	0.161	-0.267	0.365	164	0.761	2.07
Rai, 2008a	0.049	0.181	-0.305	0.404	140	0.785	1.88
Song, 2014a	0.096	0.149	-0.197	0.389	180	0.521	2.18
Bora, 2017a	0.113	0.204	-0.287	0.513	100	0.580	1.68
Wang, 2018a	0.115	0.086	-0.053	0.284	743	0.180	2.83
Zhu, 2001	0.138	0.214	-0.282	0.557	199	0.520	1.60
Xiao, 2008b	0.165	0.090	-0.013	0.342	554	0.069	2.78
Rai, 2008b	0.189	0.200	-0.204	0.581	100	0.347	1.71
Wang, 2018b	0.206	0.083	0.043	0.370	690	0.013	2.85
Larson, 2002a	0.212	0.121	-0.025	0.448	359	0.080	2.47
Li, 2008	0.219	0.085	0.051	0.386	564	0.010	2.83
Dallinga-Thie, 1996	0.230	0.099	0.036	0.425	408	0.020	2.69
Jia, 2005	0.492	0.117	0.262	0.721	307	0.000	2.51
de Luis, 2018-2	0.567	0.288	0.002	1.132	63	0.049	1.13
	-0.035	0.038	-0.109	0.039	17293	0.352	

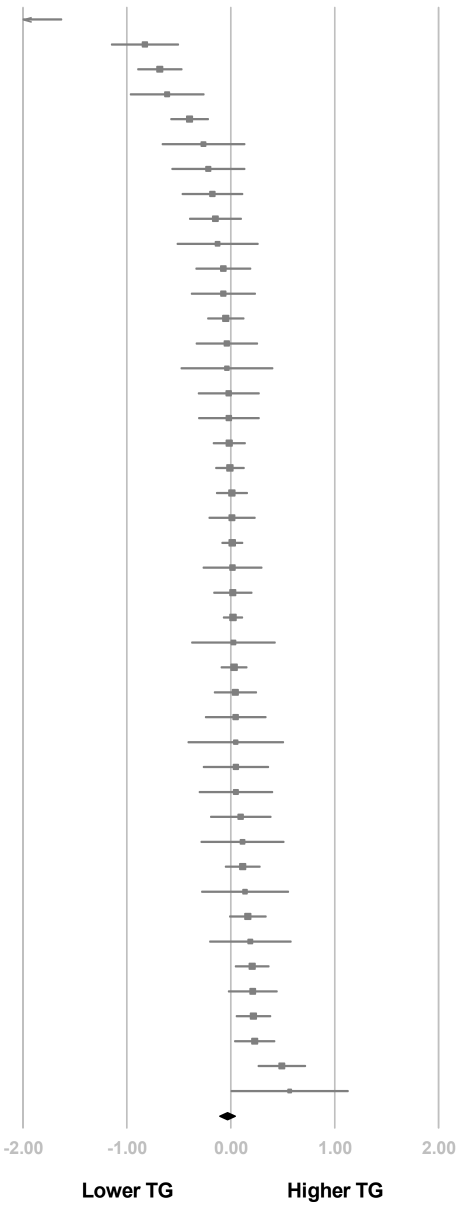

Supplementary Figure S2. (**c**) Forest plot of TG SMDs between *APOA1* rs670 genotypes

Group by / Subgroup within study	Study name	Std diff in means	Standard error	Lower limit	Upper limit	Total	p-Value	Relative weight
MetS	de Luis, 2018-1	-0.435	0.237	-0.900	0.031	82	0.067	4.09
MetS	de Luis, 2019-1a	-0.427	0.180	-0.779	-0.075	134	0.018	4.99
MetS	Bora, 2017b	-0.277	0.207	-0.683	0.128	100	0.180	4.55
MetS	Izaola, 2020	-0.244	0.204	-0.644	0.156	131	0.232	4.60
MetS	Rai, 2008a	-0.153	0.181	-0.508	0.202	140	0.399	4.97
MetS	de Luis, 2019-3b	-0.140	0.164	-0.462	0.181	174	0.392	5.25
MetS	Bai, 1996b	-0.063	0.150	-0.357	0.231	205	0.675	5.48
MetS	Bora, 2017c	-0.036	0.145	-0.321	0.248	200	0.802	5.57
MetS	Hsu, 2019	-0.021	0.048	-0.115	0.073	1751	0.657	6.89
MetS	Xiao, 2008b	-0.013	0.090	-0.190	0.164	554	0.885	6.41
MetS	Reguero, 1998	0.000	0.160	-0.313	0.313	176	1.000	5.32
MetS	Zhu, 2001	0.001	0.214	-0.419	0.420	199	0.998	4.44
MetS	Wang, 2018b	0.008	0.083	-0.155	0.172	690	0.920	6.51
MetS	de Luis, 2019-2	0.016	0.071	-0.123	0.154	902	0.827	6.66
MetS	Zou, 2003	0.115	0.200	-0.277	0.507	137	0.565	4.66
MetS	Dallinga-Thie, 1996	0.167	0.099	-0.027	0.362	408	0.092	6.29
MetS	de Luis, 2018-2	0.672	0.290	0.104	1.241	63	0.020	3.38
MetS	de Luis, 2019-3a	1.183	0.169	0.851	1.514	186	0.000	5.17
MetS	de Luis, 2019-1b	1.243	0.194	0.863	1.623	148	0.000	4.76
MetS		0.075	0.074	-0.070	0.219	6380	0.310	
non-MetS	Ramezani-Jolfaie, 2020	-0.526	0.275	-1.065	0.013	73	0.056	1.49
non-MetS	Rai, 2008b	-0.263	0.201	-0.657	0.130	100	0.190	2.38
non-MetS	Chen, 2009	-0.194	0.138	-0.465	0.077	214	0.160	3.71
non-MetS	Bai, 1996a	-0.134	0.158	-0.444	0.175	211	0.395	3.22
non-MetS	Larson, 2002b	-0.027	0.114	-0.251	0.196	372	0.810	4.43
non-MetS	Supajaree, 2022	-0.025	0.106	-0.233	0.183	355	0.812	4.69
non-MetS	Jia, 2005	-0.018	0.115	-0.244	0.208	307	0.875	4.39
non-MetS	Ordovas, 2002a	0.013	0.080	-0.143	0.169	755	0.874	5.61
non-MetS	Song, 2014a	0.019	0.149	-0.274	0.311	180	0.901	3.43
non-MetS	Ordovas, 2002b	0.022	0.077	-0.128	0.172	822	0.773	5.72
non-MetS	Xiao, 2008a	0.034	0.064	-0.092	0.159	1106	0.600	6.16
non-MetS	Costa, 2024	0.046	0.226	-0.398	0.489	81	0.840	2.01
non-MetS	de Franca, 2005	0.046	0.094	-0.139	0.231	449	0.628	5.09
non-MetS	Phillips, 2011	0.052	0.052	-0.050	0.154	1754	0.318	6.56
non-MetS	Li, 2008	0.070	0.085	-0.097	0.236	564	0.414	5.41
non-MetS	Wang, 2018a	0.080	0.086	-0.089	0.248	743	0.355	5.38
non-MetS	Song, 2014c	0.101	0.149	-0.192	0.393	181	0.499	3.43
non-MetS	Song, 2014d	0.120	0.150	-0.173	0.413	180	0.423	3.42
non-MetS	Song, 2014b	0.144	0.150	-0.149	0.437	182	0.336	3.42
non-MetS	Padmaja, 2009	0.194	0.152	-0.103	0.491	185	0.201	3.37
non-MetS	Al-Bustan, 2013	0.196	0.091	0.017	0.375	529	0.032	5.19
non-MetS	Larson, 2002a	0.224	0.121	-0.013	0.460	359	0.064	4.22
non-MetS	Bora, 2017a	0.442	0.207	0.038	0.847	100	0.032	2.29
non-MetS	Daneshpour, 2012	0.665	0.097	0.476	0.855	586	0.000	5.01
non-MetS		0.073	0.037	-0.001	0.146	10388	0.053	

Statistics for each study

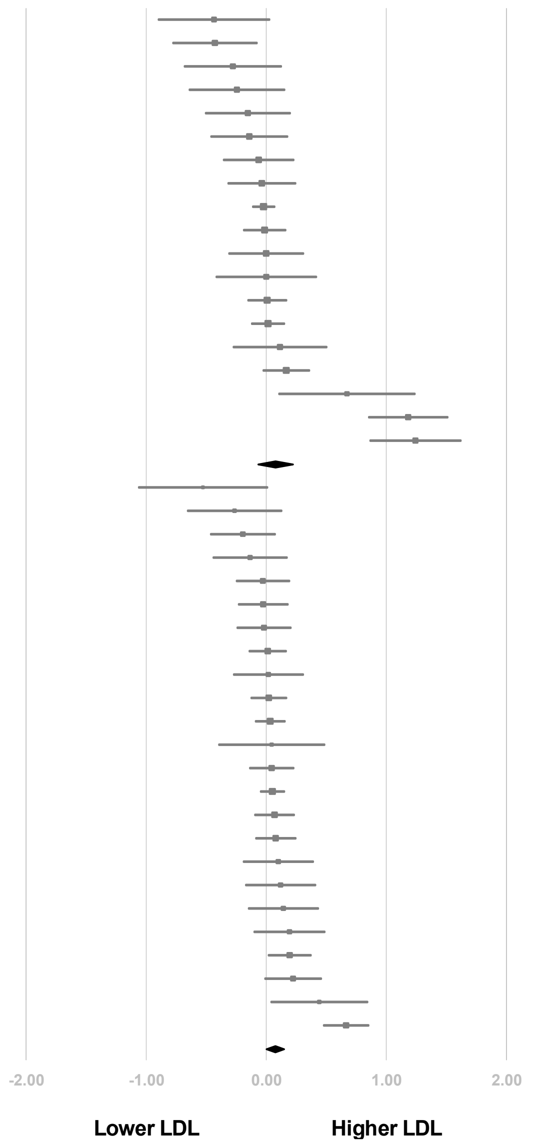

Supplementary Figure S3. (**a**) Forest plot of LDL-C SMDs between *APOA1* rs670 genotypes stratified by metabolic status (MetS vs non-MetS).

Group by Subgroup within study	Study name	Std diff in means	Standard error	Lower limit	Upper limit	Total	p-Value	Relative weight
MetS	de Luis, 2018-1	-1.028	0.248	-1.515	-0.541	82	0.000	4.31
MetS	Bora, 2017b	-0.216	0.207	-0.621	0.189	100	0.295	4.95
MetS	Chen, 2009	-0.177	0.136	-0.443	0.089	222	0.191	6.13
MetS	Rai, 2008a	-0.156	0.181	-0.511	0.199	140	0.390	5.37
MetS	Chhabra, 2005	-0.142	0.161	-0.458	0.174	164	0.380	5.71
MetS	de Luis, 2018-2	-0.067	0.284	-0.624	0.489	63	0.813	3.81
MetS	Bai, 1996b	-0.063	0.150	-0.357	0.231	205	0.675	5.89
MetS	Hsu, 2019	-0.049	0.048	-0.143	0.045	1751	0.308	7.27
MetS	Bora, 2017c	-0.000	0.145	-0.284	0.284	200	0.999	5.98
MetS	Izaola, 2020	0.052	0.203	-0.347	0.450	131	0.800	5.01
MetS	de Luis, 2019-1a	0.092	0.178	-0.257	0.441	134	0.606	5.43
MetS	de Luis, 2019-2	0.137	0.071	-0.002	0.276	902	0.053	7.05
MetS	Zhu, 2001	0.142	0.214	-0.277	0.562	199	0.506	4.84
MetS	de Luis, 2019-3b	0.158	0.164	-0.164	0.479	174	0.336	5.66
MetS	Zou, 2003	0.176	0.200	-0.216	0.568	137	0.378	5.06
MetS	Dallinga-Thie, 1996	0.311	0.100	0.115	0.506	408	0.002	6.68
MetS	de Luis, 2019-3a	0.980	0.166	0.655	1.304	186	0.000	5.63
MetS	de Luis, 2019-1b	1.067	0.190	0.695	1.440	148	0.000	5.22
MetS		0.082	0.079	-0.074	0.237	5346	0.304	
non-MetS	Ramezani-Jolfaie, 2020	-0.477	0.274	-1.015	0.061	73	0.082	0.74
non-MetS	Rai, 2008b	-0.179	0.200	-0.571	0.214	100	0.373	1.38
non-MetS	Bai, 1996a	-0.134	0.158	-0.444	0.175	211	0.395	2.22
non-MetS	Larson, 2002b	-0.027	0.114	-0.251	0.196	372	0.810	4.27
non-MetS	Reguero, 1998	0.018	0.160	-0.295	0.331	176	0.908	2.17
non-MetS	Costa, 2024	0.019	0.226	-0.425	0.462	81	0.934	1.08
non-MetS	Daneshpour, 2012	0.024	0.095	-0.161	0.210	586	0.797	6.18
non-MetS	Jia, 2005	0.027	0.115	-0.199	0.253	307	0.815	4.16
non-MetS	Phillips, 2011	0.049	0.052	-0.053	0.152	1754	0.345	20.36
non-MetS	Song, 2014a	0.061	0.149	-0.231	0.354	180	0.682	2.49
non-MetS	Supajaree, 2022	0.080	0.106	-0.128	0.288	355	0.450	4.91
non-MetS	Li, 2008	0.081	0.085	-0.085	0.248	564	0.339	7.66
non-MetS	Song, 2014c	0.105	0.149	-0.188	0.397	181	0.482	2.49
non-MetS	de Franca, 2005	0.105	0.105	-0.099	0.310	414	0.313	5.07
non-MetS	Song, 2014d	0.144	0.150	-0.149	0.437	180	0.335	2.48
non-MetS	Padmaja, 2009	0.149	0.151	-0.148	0.446	185	0.325	2.42
non-MetS	Wang, 2018a	0.161	0.086	-0.008	0.330	743	0.061	7.48
non-MetS	Al-Bustan, 2013	0.165	0.090	-0.011	0.341	549	0.067	6.88
non-MetS	Wang, 2018b	0.169	0.083	0.005	0.332	690	0.043	7.96
non-MetS	Larson, 2002a	0.198	0.121	-0.038	0.435	359	0.100	3.80
non-MetS	Song, 2014b	0.225	0.150	-0.069	0.518	182	0.134	2.47
non-MetS	Bora, 2017a	0.356	0.206	-0.048	0.759	100	0.084	1.31
non-MetS		0.085	0.024	0.038	0.131	8342	0.000	

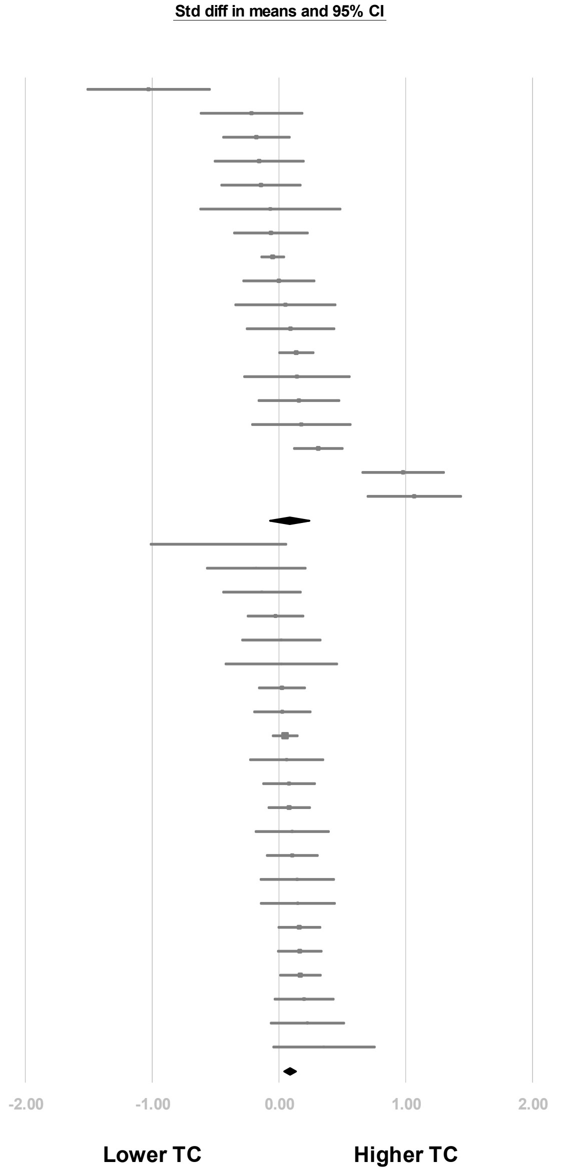

Supplementary Figure S3. (**b**) Forest plot of TC SMDs between *APOA1* rs670 genotypes stratified by metabolic status (MetS vs non-MetS).

Group by Subgroup within study	Study name	Correlation	Lower limit	Upper limit	Total	p-Value	Relative weight
MetS	de Luis, 2019-1a	-0.285	-0.429	-0.126	134	0.001	4.25
MetS	Ding, 2012	-0.192	-0.275	-0.106	483	0.000	6.93
MetS	Izaola, 2020	-0.112	-0.276	0.058	131	0.196	4.11
MetS	de Luis, 2019-1b	-0.098	-0.254	0.062	148	0.230	4.39
MetS	Zou, 2003	-0.054	-0.218	0.113	137	0.526	4.21
MetS	Chen, 2009	-0.036	-0.167	0.096	222	0.594	5.28
MetS	Bai, 1996b	-0.009	-0.145	0.127	205	0.897	5.13
MetS	de Luis, 2019-2	-0.004	-0.069	0.061	902	0.904	7.82
MetS	Bora, 2017c	0.008	-0.130	0.146	200	0.910	5.08
MetS	Hsu, 2019	0.011	-0.036	0.058	1751	0.650	8.45
MetS	Bora, 2017b	0.012	-0.181	0.206	100	0.901	3.50
MetS	de Luis, 2018-1	0.023	-0.191	0.235	82	0.838	3.08
MetS	Chhabra, 2005	0.024	-0.129	0.175	164	0.761	4.62
MetS	Rai, 2008a	0.023	-0.142	0.186	140	0.785	4.25
MetS	Zhu, 2001	0.046	-0.093	0.182	199	0.520	5.07
MetS	Xiao, 2008b	0.077	-0.006	0.159	554	0.068	7.14
MetS	Wang, 2018b	0.094	0.020	0.167	690	0.013	7.47
MetS	Dallinga-Thie, 1996	0.114	0.018	0.208	408	0.020	6.60
MetS	de Luis, 2018-2	0.244	0.006	0.456	63	0.045	2.62
MetS		-0.006	-0.053	0.040	6713	0.790	
non-MetS	Ramezani-Jolfaie, 2020	-0.700	-0.788	-0.583	73	0.000	3.00
non-MetS	Reguero, 1998	-0.363	-0.480	-0.234	176	0.000	3.73
non-MetS	Supajaree, 2022	-0.323	-0.411	-0.230	355	0.000	4.27
non-MetS	Song, 2014c	-0.088	-0.229	0.057	181	0.235	3.69
non-MetS	Chien, 2008	-0.074	-0.196	0.051	245	0.248	3.96
non-MetS	Bai, 1996a	-0.031	-0.165	0.103	211	0.651	3.83
non-MetS	Al-Bustan, 2013	-0.023	-0.106	0.061	549	0.595	4.48
non-MetS	Padmaja, 2009	-0.018	-0.161	0.125	185	0.804	3.71
non-MetS	Costa, 2024	-0.018	-0.232	0.197	81	0.871	2.78
non-MetS	Song, 2014b	-0.009	-0.153	0.136	182	0.904	3.69
non-MetS	Ordovas, 2002a	-0.007	-0.078	0.064	755	0.853	4.61
non-MetS	Ordovas, 2002b	0.005	-0.063	0.073	822	0.889	4.64
non-MetS	Larson, 2002b	0.005	-0.096	0.107	372	0.918	4.26
non-MetS	Phillips, 2011	0.007	-0.040	0.053	1754	0.782	4.83
non-MetS	Daneshpour, 2012	0.008	-0.072	0.089	586	0.838	4.51
non-MetS	Xiao, 2008a	0.015	-0.044	0.074	1106	0.620	4.73
non-MetS	de Franca, 2005c	0.021	-0.075	0.117	414	0.670	4.33
non-MetS	Song, 2014d	0.023	-0.122	0.168	180	0.753	3.68
non-MetS	Song, 2014a	0.048	-0.098	0.191	180	0.521	3.68
non-MetS	Bora, 2017a	0.055	-0.140	0.246	100	0.580	3.04
non-MetS	Wang, 2018a	0.049	-0.023	0.120	743	0.180	4.60
non-MetS	Rai, 2008b	0.094	-0.101	0.282	100	0.346	3.05
non-MetS	Larson, 2002a	0.092	-0.011	0.193	359	0.079	4.24
non-MetS	Li, 2008	0.108	0.026	0.188	564	0.010	4.49
non-MetS	Jia, 2005	0.236	0.130	0.337	307	0.000	4.15
non-MetS		-0.034	-0.088	0.021	10580	0.226	

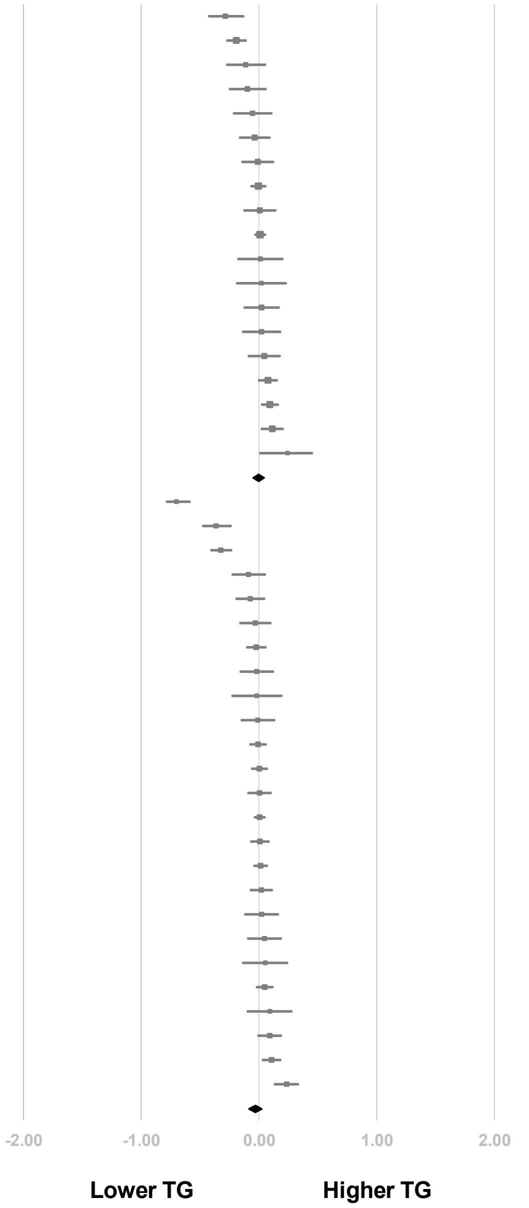

Supplementary Figure S3. (**c**) Forest plot of TG SMDs between *APOA1* rs670 genotypes stratified by metabolic status (MetS vs non-MetS).

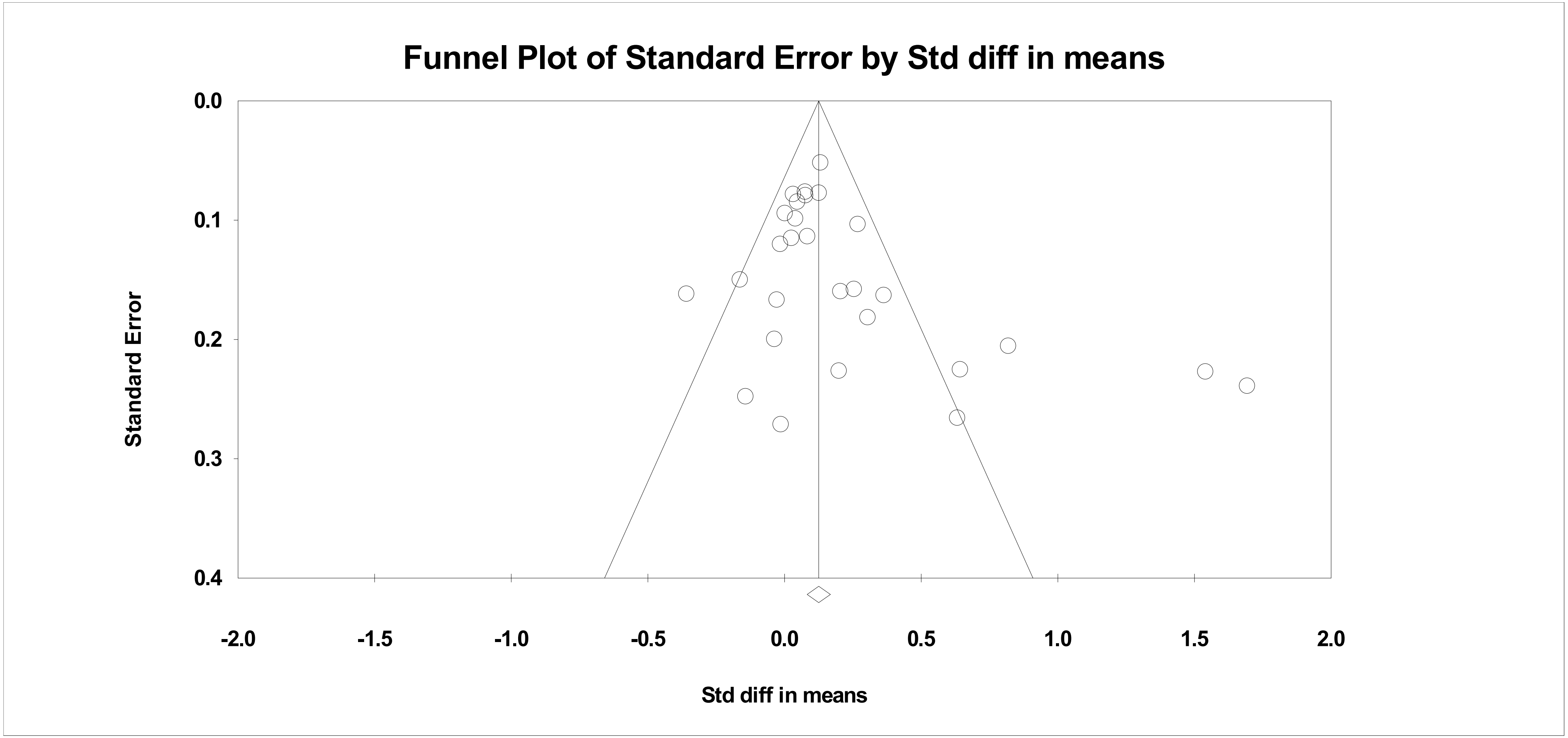

Supplementary Figure S4. (**a**) Funnel plots for publication bias of serum APOA1 SMDs between APOA1 rs670 genotypes.

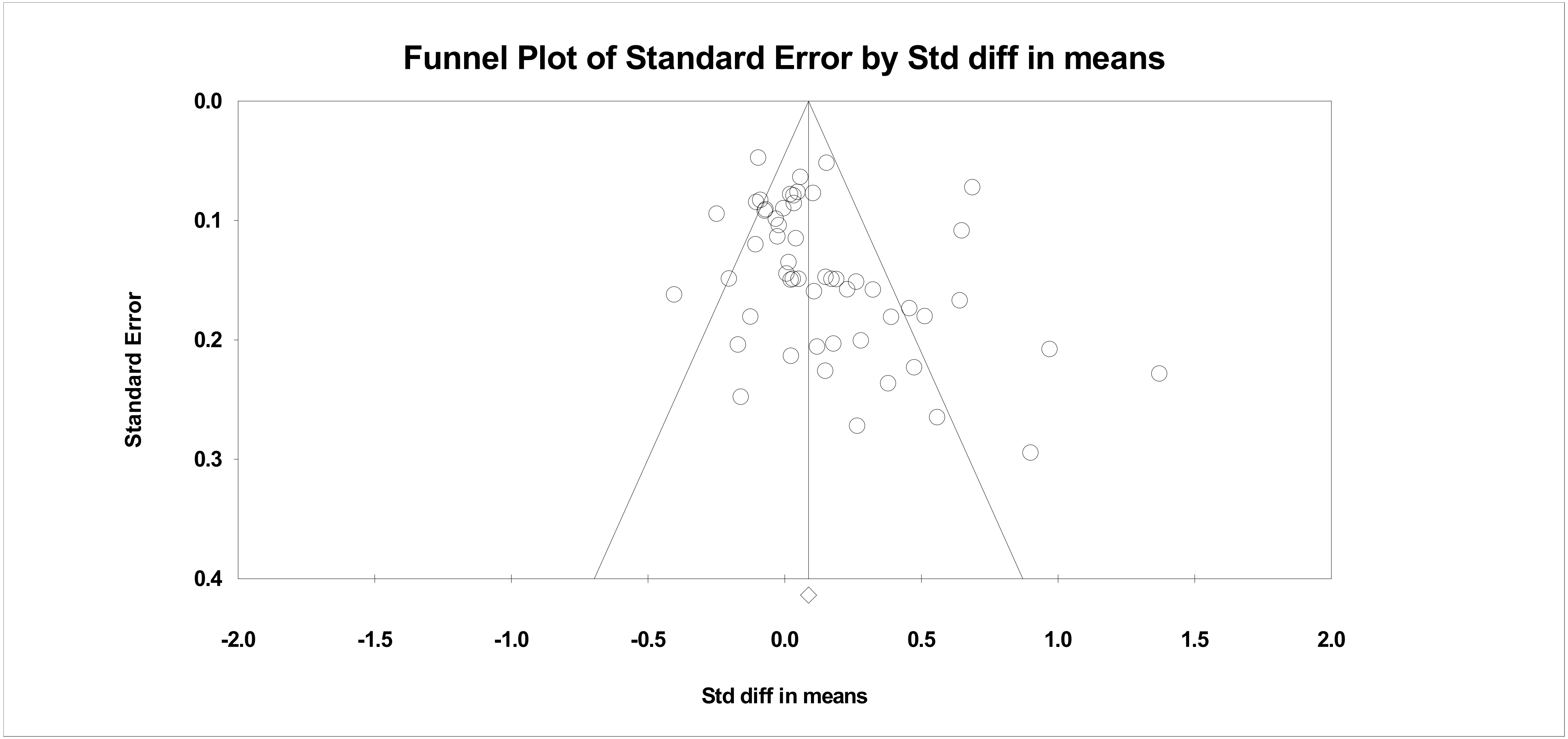

Supplementary Figure S4. (**b**) Funnel plots for publication bias of serum HDL-C SMDs between APOA1 rs670 genotypes.

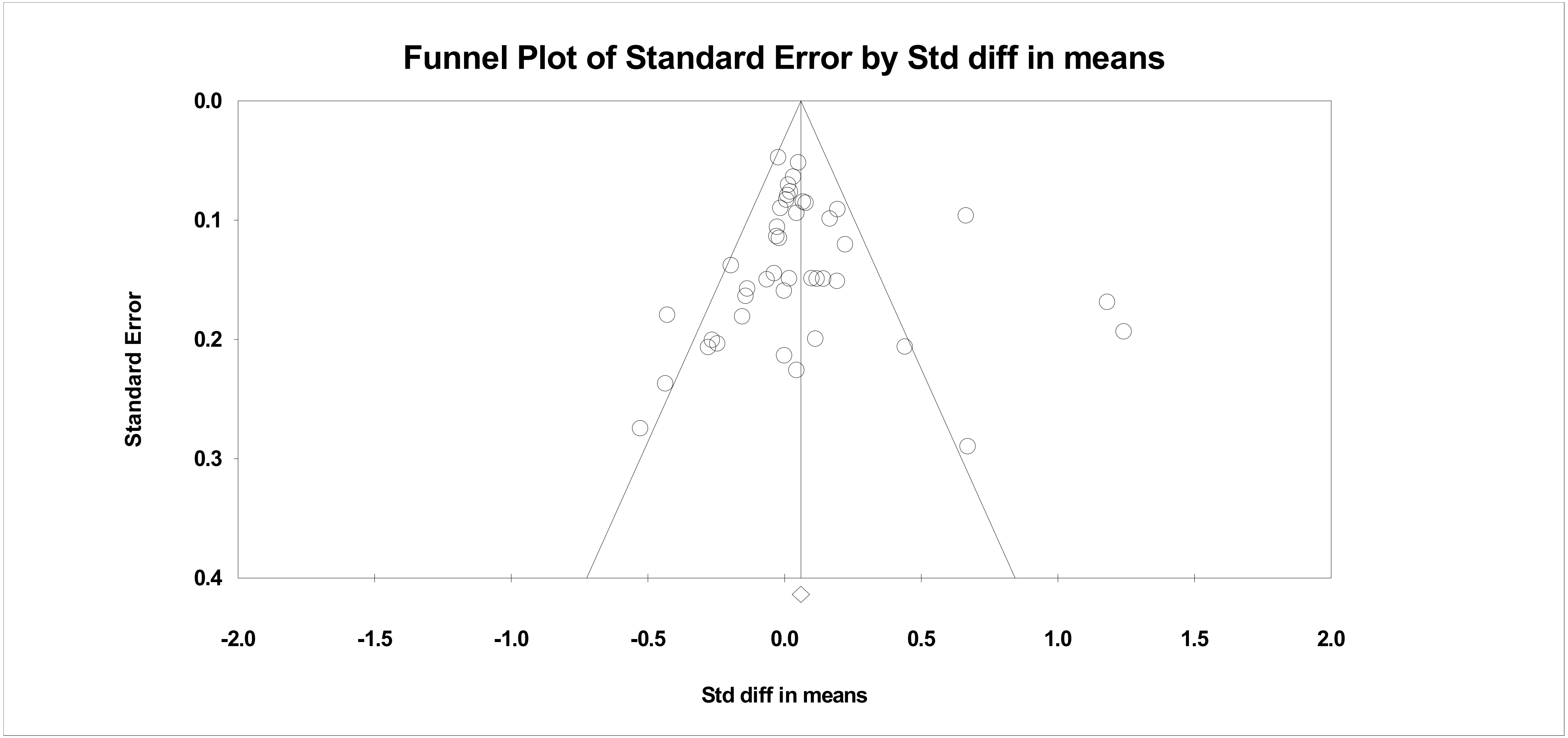

Supplementary Figure S4. (**c**) Funnel plots for publication bias of serum LDL-C SMDs between APOA1 rs670 genotypes.

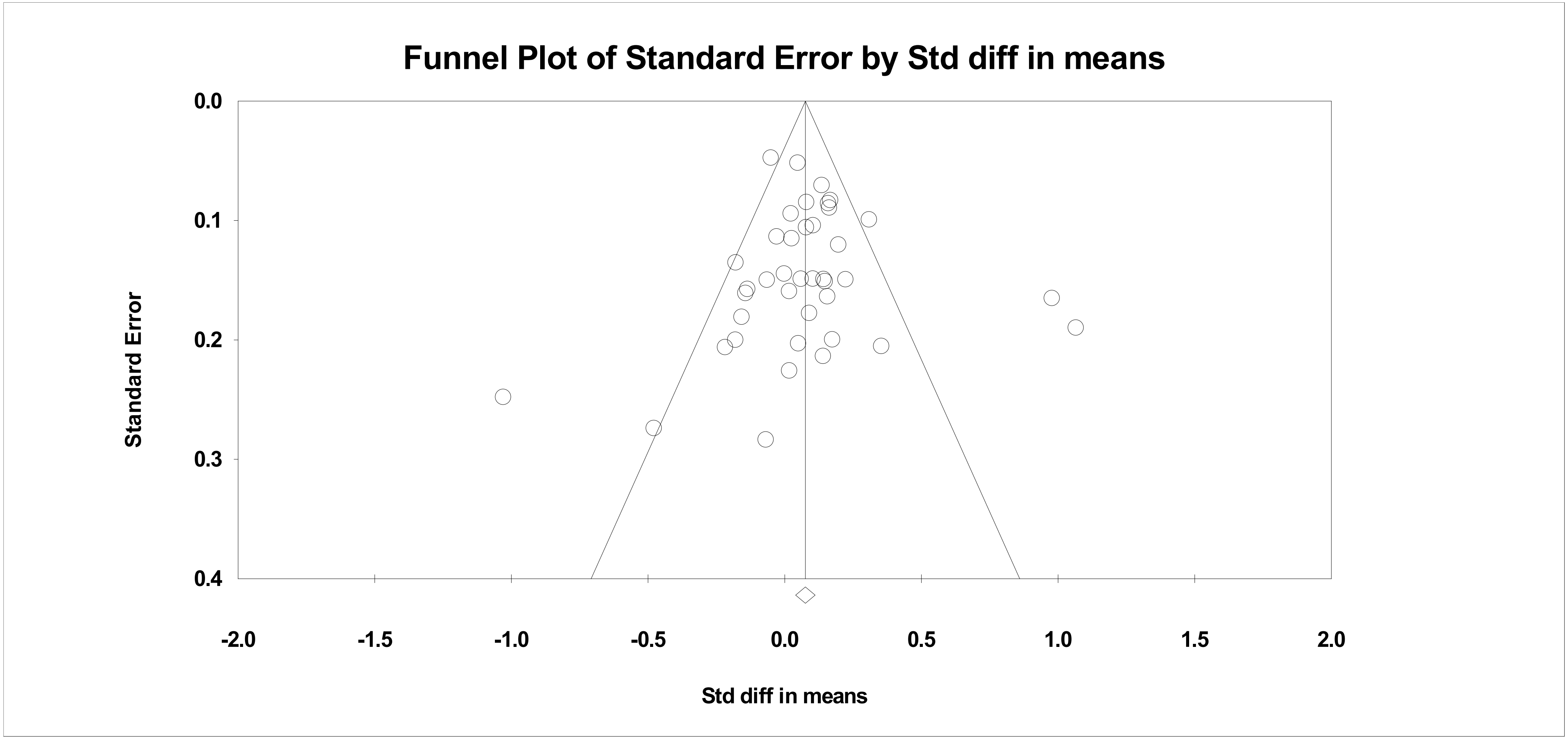

Supplementary Figure S4. (**d**) Funnel plots for publication bias of serum TC SMDs between APOA1 rs670 genotypes.

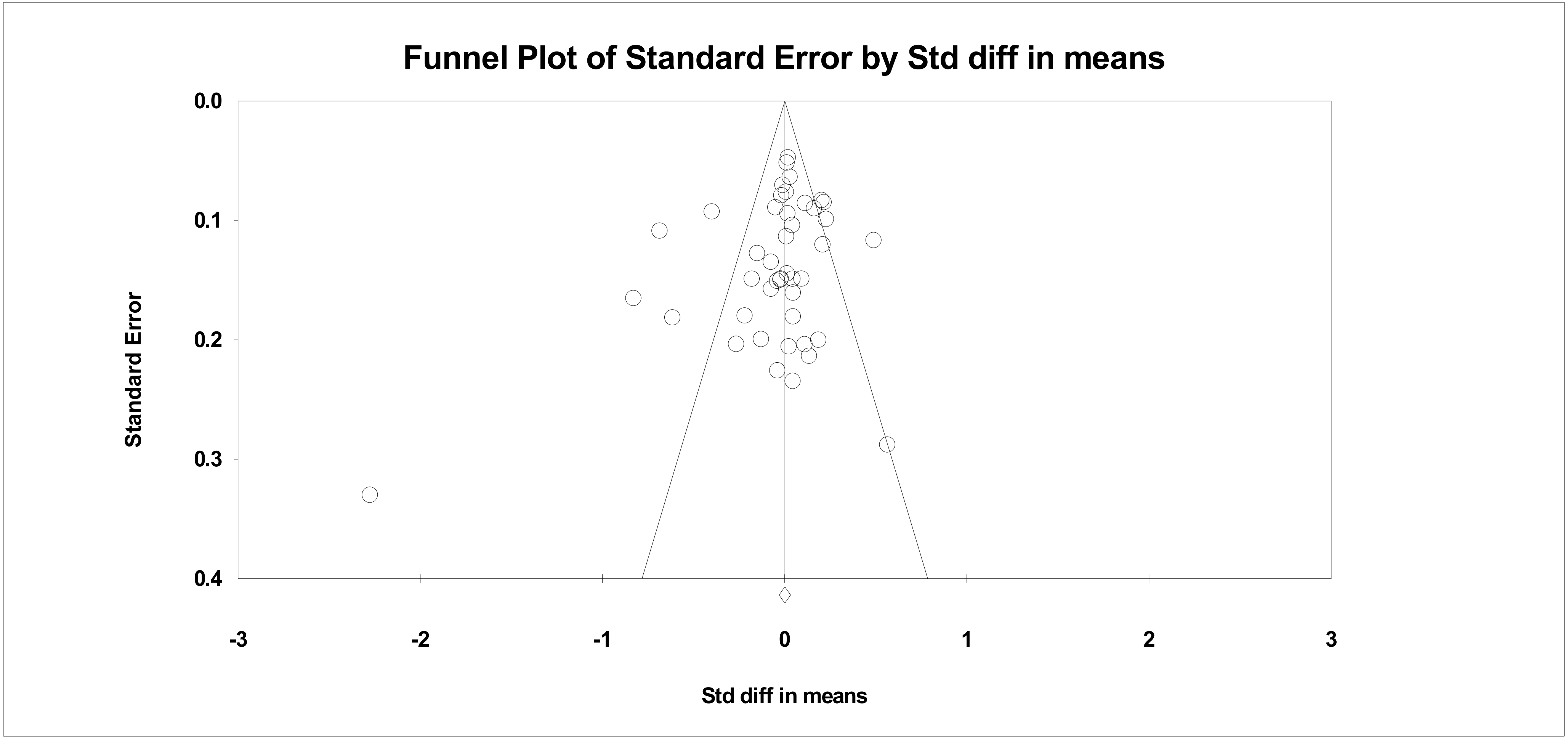

Supplementary Figure S4. (**e**) Funnel plots for publication bias of serum TG SMDs between APOA1 rs670 genotypes.

References

1. Chhabra S, Narang R, Lakshmy R, Das N. APOA1-75 G to A substitution associated with severe forms of CAD, lower levels of HDL and apoA-I among northern Indians. *Dis Markers*. 2005;21(4):169-74. doi:10.1155/2005/195078

2. Daneshpour MS, Faam B, Mansournia MA, et al. Haplotype analysis of Apo AI-CIII-AIV gene cluster and lipids level: Tehran Lipid and Glucose Study. *Endocrine*. 2012;41(1):103-10. doi:10.1007/s12020-011-9526-6

3. Bora K, Pathak MS, Borah P, Hussain MI, Das D. Association of the Apolipoprotein A-I Gene Polymorphisms with Cardiovascular Disease Risk Factors and Atherogenic Indices in Patients from Assam, Northeast India. *Balkan J Med Genet*. 2017;20(1):59-70. doi:10.1515/bjmg-2017-0002

4. Sigurdsson G, Jr., Gudnason V, Sigurdsson G, Humphries SE. Interaction between a polymorphism of the apo A-I promoter region and smoking determines plasma levels of HDL and apo A-I. *Arterioscler Thromb*. 1992;12(9):1017-22. doi:10.1161/01.atv.12.9.1017

5. de Franca E, Alves JG, Hutz MH. APOA1/C3/A4 gene cluster variability and lipid levels in Brazilian children. *Braz J Med Biol Res*. 2005;38(4):535-41. doi:10.1590/s0100-879x2005000400006

6. Rai TS, Khullar M, Sehrawat BS, et al. Synergistic effect between apolipoprotein E and apolipoprotein A1 gene polymorphisms in the risk for coronary artery disease. *Mol Cell Biochem*. 2008;313(1-2):139-46. doi:10.1007/s11010-008-9751-3

7. Larson IA, Ordovas JM, Barnard JR, et al. Effects of apolipoprotein A-I genetic variations on plasma apolipoprotein, serum lipoprotein and glucose levels. *Clin Genet*. 2002;61(3):176-84. doi:10.1034/j.1399-0004.2002.610302.x

8. Li Y, Yin R, Zhou Y, et al. Associations of the apolipoprotein A-I gene polymorphism and serum lipid levels in the Guangxi Hei Yi Zhuang and Han populations. *Int J Mol Med*. 2008;21(6):753-64.

9. Hsu LC, Hsu LS, Lee TH. Association of apolipoprotein A1 and A5 polymorphisms with stroke subtypes in Han Chinese people in Taiwan. *Gene*. 2019;684:76-81. doi:10.1016/j.gene.2018.10.050

10. Wang X, Guo H, Li Y, et al. Interactions among genes involved in reverse cholesterol transport and in the response to environmental factors in dyslipidemia in subjects from the Xinjiang rural area. *PLoS One*. 2018;13(5):e0196042. doi:10.1371/journal.pone.0196042

11. Ding Y, Zhu MA, Wang ZX, Zhu J, Feng JB, Li DS. Associations of polymorphisms in the apolipoprotein APOA1-C3-A5 gene cluster with acute coronary syndrome. *J Biomed Biotechnol*. 2012;2012:509420. doi:10.1155/2012/509420

12. Al-Bustan SA, Al-Serri AE, Annice BG, Alnaqeeb MA, Ebrahim GA. Re-sequencing of the APOAI promoter region and the genetic association of the -75G > A polymorphism with increased cholesterol and low density lipoprotein levels among a sample of the Kuwaiti population. *BMC Med Genet*. 2013;14:90. doi:10.1186/1471-2350-14-90

13. Dallinga-Thie GM, Bu XD, van Linde-Sibenius Trip M, Rotter JI, Lusis AJ, de Bruin TW. Apolipoprotein A-I/C-III/A-IV gene cluster in familial combined hyperlipidemia: effects on LDL-cholesterol and apolipoproteins B and C-III. *J Lipid Res*. 1996;37(1):136-47.

14. Xiao J, Zhang F, Wiltshire S, et al. The apolipoprotein AII rs5082 variant is associated with reduced risk of coronary artery disease in an Australian male

population. *Atherosclerosis*. 2008;199(2):333-9. doi:10.1016/j.atherosclerosis.2007.11.017
15. Chen ES, Mazzotti DR, Furuya TK, et al. Apolipoprotein A1 gene polymorphisms as risk factors for hypertension and obesity. *Clin Exp Med*. 2009;9(4):319-25. doi:10.1007/s10238-009-0051-3
16. Talmud PJ, Ye S, Humphries SE. Polymorphism in the promoter region of the apolipoprotein AI gene associated with differences in apolipoprotein AI levels: the European Atherosclerosis Research Study. *Genet Epidemiol*. 1994;11(3):265-80. doi:10.1002/gepi.1370110305
17. Bai H, Saku K, Liu R, Jimi S, Arakawa K. Analysis of a new polymorphism in the human apolipoprotein A-I gene: association with serum lipoprotein levels and coronary heart disease. *J Cardiol*. 1996;28(4):207-12.
18. Zhu H, Liu Y, Bai H, Liu B-W. Apolipoprotein A_Ⅰ Gene MspI Polymorphism in Relation to Endogenous Hypertriglyceridemia in Chinese Population. *Editorial Office of Chinese Journal of Arteriosclerosis*. 2001;9(4):332-336.
19. Song YY, Gong RR, Zhang Z, et al. [Effects of apolipoprotein A1 gene rs670 and rs5069 polymorphisms on the plasma lipid profiles in healthy adolescents with different body mass index]. *Zhongguo Yi Xue Ke Xue Yuan Xue Bao*. 2014;36(4):369-76. doi:10.3881/j.issn.1000-503X.2014.04.005
20. Ordovas JM, Corella D, Cupples LA, et al. Polyunsaturated fatty acids modulate the effects of the APOA1 G-A polymorphism on HDL-cholesterol concentrations in a sex-specific manner: the Framingham Study. *Am J Clin Nutr*. 2002;75(1):38-46. doi:10.1093/ajcn/75.1.38
21. Jia L, Bai H, Fu M, Xu Y, Yang Y, Long S. Relationship between plasma HDL subclasses distribution and apoA-I gene polymorphisms. *Clin Chim Acta*. 2005;360(1-2):37-45. doi:10.1016/j.cccn.2005.04.004
22. Reguero JR, Cubero GI, Batalla A, et al. Apolipoprotein A1 gene polymorphisms and risk of early coronary disease. *Cardiology*. 1998;90(3):231-5. doi:10.1159/000006849
23. Costa M, Silva M, Viana G, et al. Single nucleotide polymorphisms in apolipoprotein A-I (rs670) and B (rs693) associated with serum lipoproteins and endothelial activation in COVID-19 outpatients. *Brazilian Journal of Pharmaceutical Sciences*. 2024;60doi:10.1590/s2175-97902024e24015
24. Phillips CM, Goumidi L, Bertrais S, et al. Gene-nutrient interactions and gender may modulate the association between ApoA1 and ApoB gene polymorphisms and metabolic syndrome risk. *Atherosclerosis*. 2011;214(2):408-14. doi:10.1016/j.atherosclerosis.2010.10.029
25. Izaola O, Primo D, Gomez Hoyos E, Lopez Gomez JJ, Ortola A, de Luis D. Association of rs670 variant of APOA1 gene with lipid profile and insulin resistance after 9 months of a high protein/low carbohydrate vs a standard hypocaloric diet. *Clin Nutr*. 2020;39(4):988-993. doi:10.1016/j.clnu.2019.04.030
26. Padmaja N, Ravindra Kumar M, Adithan C. Apolipoprotein AI and Apolipoprotein B gene polymorphisms and lipid profile in Tamilian population. *Ann Hum Biol*. 2009;36(2):220-7. doi:10.1080/03014460802604268
27. Ramezani-Jolfaie N, Aghaei S, Yazd EF, et al. Association of rs670 variant of APOA-1 gene with cardiometabolic markers after consuming sesame, canola and sesame-canola oils in adults with and without type 2 diabetes mellitus. *Clin Nutr ESPEN*. 2020;38:129-137. doi:10.1016/j.clnesp.2020.05.016
28. de Luis Román DA, Izaola Jáuregui O, Primo D, Aller R. [Influence of rs670 variant of APOA1 gene on serum HDL response to an enriched-polyunsaturated vs. an enriched-monounsaturated fat hypocaloric diet]. *Nutr Hosp*. 2019;36(6):1288-1295. doi:10.20960/nh.02390

29. de Luis DA, Izaola O, Primo D, Aller R. Implication of the rs670 variant of APOA1 gene with lipid profile, serum adipokine levels and components of metabolic syndrome in adult obese subjects. *Clin Nutr*. 2019;38(1):407-411. doi:10.1016/j.clnu.2017.12.007
30. de Luis DA, Izaola O, Primo D, Aller R. Role of rs670 variant of APOA1 gene on lipid profile, insulin resistance and adipokine levels in obese subjects after weight loss with a dietary intervention. *Diabetes Res Clin Pract*. 2018;142:139-145. doi:10.1016/j.diabres.2018.05.040
31. Wang XL, Badenhop R, Humphrey KE, Wilcken DE. New MspI polymorphism at +83 bp of the human apolipoprotein AI gene: association with increased circulating high density lipoprotein cholesterol levels. *Genet Epidemiol*. 1996;13(1):1-10. doi:10.1002/(sici)1098-2272(1996)13:1<1::Aid-gepi1>3.0.Co;2-d
32. Jeenah M, Kessling A, Miller N, Humphries S. G to A substitution in the promoter region of the apolipoprotein AI gene is associated with elevated serum apolipoprotein AI and high density lipoprotein cholesterol concentrations. *Mol Biol Med*. 1990;7(3):233-41.
33. Supajaree P, Chanprasertyothin S, Chattranukulchai Shantavasinkul P, Sritara P, Sirivarasai J. Association between ApoA1 Gene, Plasma Lipid Profile, hsCRP Level, and Risk of Arterial Stiffness in Thai Elderly. *Adv Prev Med*. 2022;2022:4930033. doi:10.1155/2022/4930033
34. de Luis D, Izaola O, Primo D, Aller R. Role of rs670 variant of APOA1 gene on metabolic response after a high fat vs. a low fat hypocaloric diets in obese human subjects. *J Diabetes Complications*. 2019;33(3):249-254. doi:10.1016/j.jdiacomp.2018.10.015
35. de Luis DA, Pacheco D, Izaola O, Primo D, Aller R. The association of the rs670 variant of APOA1 gene with insulin resistance and lipid profile in morbid obese patients after a biliopancreatic diversion surgery. *Eur Rev Med Pharmacol Sci*. 2018;22(23):8472-8479. doi:10.26355/eurrev_201812_16547
36. Zou Y, Hu D, Yang X, et al. Relationships among apolipoprotein A1 gene polymorphisms, lipid levels and coronary atherosclerosis disease. *Chin Med J (Engl)*. 2003;116(5):665-8.
37. Dawar R, Gurtoo A, Singh R. Apolipoprotein A1 gene polymorphism (G-75A and C+83T) in patients with myocardial infarction: a pilot study in a north Indian population. *Am J Clin Pathol*. 2010;134(2):249-55. doi:10.1309/ajcpkptxq3qn1ifg
38. Bora K, Pathak MS, Borah P, Hussain MI, Das D. Single nucleotide polymorphisms of APOA1 gene and their relationship with serum apolipoprotein A-I concentrations in the native population of Assam. *Meta Gene*. 2016;7:20-7. doi:10.1016/j.mgene.2015.10.005
39. Swanson CR, Li K, Unger TL, et al. Lower plasma apolipoprotein A1 levels are found in Parkinson's disease and associate with apolipoprotein A1 genotype. *Mov Disord*. 2015;30(6):805-12. doi:10.1002/mds.26022
40. Chien KL, Fang WH, Wen HC, et al. APOA1/C3/A5 haplotype and risk of hypertriglyceridemia in Taiwanese. *Clin Chim Acta*. 2008;390(1-2):56-62. doi:10.1016/j.cca.2007.12.014.
